# Modelling the impact of migration on FGM/C prevalence and risk in immigrant and diaspora populations

**DOI:** 10.1371/journal.pone.0360038

**Published:** 2026-10-13

**Authors:** Sean Callaghan

**Affiliations:** School of Criminology, University of Leicester, Fielding Johnson Building, Leicester, United Kingdom; University at Buffalo, UNITED STATES OF AMERICA

## Abstract

Previous estimates of the number of women and girls impacted by female genital mutilation and cutting (FGM/C) in the United States (US) failed to account for the impact of migration and acculturation. Similar studies in Europe applied a standard factor to estimate the impact across the entire immigrant population. Several of the resultant European estimates offered two scenarios: no impact, in which prevalence remained unaffected by migration and acculturation, and partial impact, in which risk was assumed to drop by 50% for native-born children. Each of these previous studies was based on adaptations of the extrapolation method, which produces estimates based on the product of three input variables: the immigrant population in the country of residence, the prevalence in the country of origin, and the mitigating impact of migration and acculturation. This paper offers several methodological innovations to better estimate the impact of migration and applies the results to the US. It develops methods to account for selective migration, the mitigating impact of legislation and law enforcement, and it considers the age of migration and the age of cutting to estimate the impact of migration timing on the likelihood that individuals were cut prior to migration. Using these innovations to model the impact of migration on the population in the US suggested that previous studies had underestimated the number of women living with FGM/C, while overestimating the number of girls who were at risk.

## Introduction

The World Health Organisation (WHO) define female genital mutilation/cutting (FGM/C) as the “partial or total removal of external female genitalia or other injury to the female genital organs for non-medical reasons” [1]. Evidence suggests that the practice is prevalent in indigenous and immigrant populations in at least 94 countries [2]. While most survivors are African [3], population sizes in countries of practice vary dramatically, resulting in a concentration of impacted populations in Indonesia, Nigeria and Egypt, which accounted for more than half of the 4.3 million girls at risk each year [4], almost all of whom are cut before the age of 15 [5].

The method most often used for estimating the scale and distribution of the FGM/C-impacted population in diaspora contexts is the Extrapolation-of-FGM/C-Countries’-Prevalence-Data method [6]. The extrapolation method was originally proposed by Yoder *et al*. [7] for estimating the number of women living with FGM/C in Africa. At the time, Yoder *et al*. [7] argued that the extrapolation method they used was ill-suited to the estimation of the population living with FGM/C in immigrant contexts, pointing to selective migration, intra-country ethnic diversity, and declining prevalence in countries of origin as issues that the model did not address and which would likely impact the accuracy of the extrapolated results. Notwithstanding these concerns, Van Baelen *et al.* [8] applied the method in Europe. In this form, the extrapolation method consisted of two variables: FGM/C prevalence (Pr) in the country of origin and the first-generation immigrant population (p) in the country under examination. The product of these variables produced an estimate of the number of immigrants already living with FGM/C. The European Institute for Gender Equality (EIGE) adapted the extrapolation method [9] to estimate the number of first and second-generation girls who were at risk of FGM/C in Europe. In so doing, the EIGE added a third variable to the equation: an estimation of the impact of migration and acculturation (m). In the EIGE method, two scenarios are calculated: in the high-risk scenario, migration and acculturation are assumed to have no impact on risk (m = 0), while in the low-risk scenario, native-born girls are considered to be at half the risk (m = 0.5) of their foreign-born counterparts.

Kawous *et al.* [10], Nestlinger *et al.* [11] and Stegner et al. [12] effectively combined these two approaches: to calculate the number of women and girls living with FGM/C, they used van Baelen et al.’s [8] approach in which migration and acculturation are not considered, while they turned to the EIGE method [9] to estimate the number of girls at risk. Stegner et al. [12] followed the EIGE assumption that the risk to native-born girls was half that of foreign-born girls (m = 0.5). On the other hand, Kawous *et al.* [10] conducted a sensitivity analysis setting m at various levels between 0 and 1, whereas Nestlinger *et al.* [11] calculated country-specific values for m based on survey data. While there were additional nuances in each of these studies, their combined approach to calculating the potentially impacted population (both those living with FGM and those at risk) could broadly be expressed in the following extrapolation formula:


$$\begin{array}{c} \text{IP}=\sum_{\text{n}=\text{1}}^{\text{c}}\left({\text{Pr}}_{(\text{x},\text{y}),\text{n}}\;\times \;{\text{p}}_{(\text{x},\text{y}),\text{n}}\;\times \;(\text{1}-m_{n}\right)) \end{array}$$

Where:

IP = the population impacted by FGM/C (both living with and at risk)

${\text{Pr}}_{(\text{x},\text{y}),\text{n}}$ = the FGM/C prevalence disaggregated by five-year age cohorts (x,y) in a specific country-of-origin (n)

${\text{p}}_{(\text{x},\text{y}),\text{n}}\;$ = the first- and second-generation study population in the country-of-residence associated with a specific country-of-origin (n) disaggregated by five-year age cohorts (x,y)

(x,y) = five-year age cohorts 0-4, 5-9, 10-14, 15-19, 20-24 etc.

$m_{n}$ = a composite variable indicating the migration and acculturation impact in a range from 0 (no impact) to 1 (total impact)

c = the number of countries of origin under investigation

Previous studies in the United States (US), on the other hand, were based on an even simpler formula: Jones *et al.* [13] simply multiplied the study population by the national average prevalence in countries of origin to establish the total number of women and girls living with or at risk of FGM/C. Goldberg et al. [14] split the study population at age 20 and applied the 15–19 prevalence to those younger than 20 and the national average to the rest. Neither made any attempt to consider the impact of migration and acculturation.

In the formulation above, the single variable (m) models the impact of both migration and acculturation. This paper will explore the profound heterogeneity hidden within that single variable (m). However, in so doing, the paper will seek to model the impact of migration alone. While migration clearly also influences acculturation strategies and experiences, modelling the impact of acculturation is beyond the scope of this study.

There are several aspects of migration that have a direct impact on the prevalence and risk of FGM/C in immigrant and diaspora communities. First, selective migration determines the composition of the immigrant population. Second, migration timing and its interplay with the age of FGM/C determine an individual’s FGM/C status at the time of migration. Third, migration exposes individuals to new constraints, such as legislation and law enforcement. Fourth, transnational travel and vacation visits expose both native-born and foreign-born girls to risk during visits to their parents’ countries of origin.

### The impact of selective migration

The impact of selective migration must be considered throughout the extrapolation equation. Applying De Haas’s [15] aspirations and capabilities framework to the diaspora context suggests that there are two primary pathways for relocation from FGM/C-practicing communities: immigration (voluntary mobility) or refugee resettlement (involuntary mobility). Whether for education, employment or family reunification, accessing voluntary mobility options into the US or Europe requires a level of economic and educational status that would tend to exclude less privileged individuals and communities [15–17]. This observation supports Ortensi *et al*.’s [18,19] *selection hypothesis,* which accounts for the impact of socio-economic-driven selective migration by moderating prevalence to account for a more educated, wealthier, and urban immigrant population. When it comes to involuntary migration, refugee resettlement routes are less obviously driven by those three demographic indicators of status. However, it can be argued that the differential mobility options available to people facing forced displacement segment populations into internally displaced persons (IDPs) or refugees along very similar lines [20]. Since refugee resettlement draws its participants from the latter group, it can be argued that Ortensi *et al.’s* assertion holds in relative terms. Likewise, a similar pattern can be observed among asylum seekers [21].

Selective migration is not only a factor of socio-economic status, but it also shapes the ethnic composition of the immigrant population as observed by Yoder *et al.* [7]. For example, when one considers the prevalence of FGM/C across the Horn of Africa, it can be seen that prevalence rates in eastern Ethiopia and north-eastern Kenya are essentially the same as those in neighbouring Somalia [22]. Each of those regions shows a prevalence of FGM/C above 90%, which is significantly higher than the national average for Kenya and Ethiopia, where prevalence is 14.8% and 65.2%, respectively [23]. It is known that the populations in these border areas are predominantly ethnic Somali, which drives the higher-than-national-average prevalence in these regions [22]. Selecting the study population based on place of birth rather than ancestry, as Jones *et al.* [13] and Goldberg *et al.* [14] did, masks the impact of selective migration. In this case, Somali’s born in Kenya and Ethiopia would have been misclassified and, as a result, assumed to be much less at risk than was likely the case.

Furthermore, without considering the impact of selective migration on prevalence, the extrapolation method falsely assumes that the immigrant population is fully representative of the population in the country of origin. For example, Ukoha [24] found that FGM/C prevalence among Nigerian immigrants in Texas was 46% – more than double the national average of 19.2% in Nigeria [25]. Likewise, Behrendt [26] and Farina and Ortensi [27] found that 45% and 74.3% of Nigerian women in their studies in Germany and Italy had undergone FGM/C. Since the prevalence in Nigeria varies significantly by ethnic origin and geographic location (state) [25], these results suggest that ethnic-specific selective migration was playing a significant role in these communities. A closer look at the ethnicity of the populations in these three studies confirms that suggestion. The population in Texas identified as Igbo, while the German study found that the majority of participants identified as Igbo or Yoruba. Prevalence among the Yoruba (54.5%) and Igbo (45.2%) are consistent with the higher figures observed in those two studies [25]. On the other hand, Farina and Ortensi [27] found that the majority of the Nigerian population in their Italian study originated from Edo State, which they note had a prevalence of over 50% at the time. While the findings of these three studies cannot be extrapolated to the entire Nigerian immigrant population, they are suggestive of an ethnic-specific selective migration pattern that should be considered.

Similarly, there are potential selective migratory impacts evident in the Egyptian community in the US in particular. The Christian population of Egypt is estimated at approximately 10% [28], while Coptic Christians make up a substantial majority of the Egyptian diaspora in the US [29]. The significance of this selective migratory pattern on the prevalence of FGM/C in the Egyptian community in the US is profound, with Christian women almost three times more likely to believe that FGM/C should be discontinued than their Muslim counterparts – at a ratio of 76.1% to 29.5% respectively [30]. Within that context, Agboli *et al.* suggest that “[the] migration context appears to be a favourable platform for the change of views towards the practice of FGM” [31]. This view is shared by others who point to the opportunity that migration offers for reflection on one’s cultural practice [32–35]. Agboli *et al.* [31] proposed a five-stage model of attitude change that is triggered by migration. In their model, women shift from *Emotion Suppression,* in which they conform to community expectations prior to migration, through a process of *Awakening* that is precipitated by a series of turning points – moments of awareness – post migration, which trigger questioning of FGM/C. A *Clash* follows as women experience internal conflicts of loyalty between community norms and their well-being (especially when protecting their daughters). In time, women actively reconstruct their identity by reconciling traumatic memories and rejecting harmful norms, leading to a *Re-appropriation of Self* and eventually *Reconciliation* as they forgive their mothers and maintain cultural ties, while rejecting the practice of FGM/C, mirroring Berry’s [36] integration acculturation strategy. While the process that Agboli *et al.* [31] describes could be applied to any immigrant population, the attitudinal data and the selective migratory trend within the Egyptian population suggests a special case.

### The impact of migration timing

Kawous *et al.* [10] proposed a method by which the immigrant population could be segmented by considering the age of migration. In their calculation, those who migrated after the age of 19 were believed to have been cut prior to migration at the prevailing rate of prevalence in their country of origin. Those who migrated below the age of 20 were considered to be at risk. While Kawous et al. [10] acknowledged the impact of migration timing in their analysis, they ignored the heterogeneity of the age of cutting [5] across diverse populations.

A more accurate approach would consider the typical age of cutting in a specific community when determining the potential FGM/C status of an immigrant, similar to that proposed by the EIGE [9], since the age of FGM/C differs from one community to another, spanning a range from a few weeks after birth through to late teens. Furthermore, analysis of prevalence data suggests that the risk of FGM/C in any given community is often confined to a narrow age range [5]. For girls in communities that cut within the first few months of life – including communities in Nigeria and Yemen [23] – the risk of FGM/C is highest before the girl’s first birthday and is essentially non-existent in her teen years. If a girl from one of these communities reaches puberty without being cut, it is improbable that she would be at risk of FGM/C in the future. Likewise, girls growing up in communities that cut around puberty, including those from the Maasai and Samburu communities of Kenya [37], would almost certainly not be at risk as toddlers. Considering the age of cutting and the age of migration allows one to establish the likely FGM/C status of a girl when she migrates.

The age at which people migrate not only determines their FGM/C status at the time of migration, but since time in the diaspora context appears to be a factor in immigrants’ views of FGM/C [31], it also determines the age at which an individual is exposed to the destination country’s culture and laws. A study of Somali immigrants in London [38] found that age of migration and exposure to the majority culture in the United Kingdom (UK) were both indicators of risk reduction. Those who immigrated to the UK below the typical age of cutting were less likely to be cut (42%) than those who arrived after the age of cutting (91%). Morison *et al.* [38] found that while 18% of women and 43% of men indicated their intention to have their daughters cut, support for continuing the practice was skewed towards those who arrived in the UK at an older age. Likewise, in Sweden and Norway, new arrivals were less likely to support complete abandonment than those who had been in the country for at least five years. Among established Somali immigrants in Sweden, 83% supported complete abandonment and 17% supported Sunna circumcision. On the other hand, 4% of newly arrived Somalis supported Pharaonic FGM/C, 37% Sunna circumcision and only 59% complete abandonment [39]. Similarly, the 70% support for abandonment of FGM/C among Somali communities in Norway skewed towards those who had been in the country longer [40]. These empirical studies resonate with the wider literature in which Gindelsky [41] and Bleakley and Chin [42] point to a significantly higher likelihood of assimilation if an individual migrated by age 9.

### The structural constraints of legislation and law enforcement

Migration also exposes individuals to structural constraints that were not present in their countries of origin, including the dampening effects of legislation and law enforcement on FGM/C risk, as suggested by Kawous *et al.* [10]. Framing that legislative response in terms of Deterrence Theory [43], relocation to the US or Europe seems to significantly shift immigrants’ perspectives, with law enforcement and child protection policy perceived as creating a level of certainty, severity and celerity that was not present prior to migration [44]. As Kaplan *et al.* noted, “[t]he main strategy used by migrants to avoid circumcision of their daughters is to refer to the law in the destination country and the consequences it would have for the whole family” [45]. However, the level of perceived deterrence seems to be out of step with reality. While FGM/C has been criminalised in the US and across Europe for decades, very few cases have been brought to court. This seems to undermine the perspective that legislation and law enforcement provided an effective deterrence in the case of FGM/C.

More than deterrence, Kaplan *et al.* speak to the labelling impact of law enforcement, noting that “families have been criminalised and persecuted for planning or having carried out a trip to the country of origin” [45]. Likewise, Tesfamariam (quoted by Berer) spoke to the counterproductive impact of labelling, in her claims that “vilifying and punishing immigrant parents who already live on the margins of society only pushes them further away from mainstream society, making them cling to their own cultures and traditions more tightly” [46].

If anti-FGM/C legislation, both in countries of origin and countries of residence, has failed to provide an effective deterrence to FGM/C, and has instead often resulted in labelling, as is suggested by Berer [46], what then is the way forward? In arguing against a decriminalised approach, Hurn and Pinder assert that “[t]he lack of a law also makes a strong statement” [47] and signals a tacit acceptance of the practice. On the other hand, as Tesfamariam noted (as quoted by Berer), “[d]emanding an instant, law-enforced end to FGM does nothing to change the hearts and minds of communities” [46]. Instead of criminalisation, Berer calls for “community-led educational information and more support for dialogue within FGM-practising communities” [46]. In so doing, Berer calls for policy frameworks that are more focused on prevention. Central to the approach is the recognition that communities are unique and that interventions need to be developed and implemented in partnership and dialogue with the community and not imposed on the community, allowing migration to operate as a turning point in views towards the practice.

Balancing these perspectives speaks not only to the question of criminalisation, but more specifically to how legislation is framed. In their 7P framework, Mergaert *et al.* [48] proposed a policy landscape that balanced prevention initiatives and the provision of services to survivors on the one hand, with child protection and criminal prosecution on the other.

## Methodology

### Data sources

The method proposed by Callaghan [49] was used to calculate the age-disaggregated prevalence data used in the analysis in this paper. Most of the input prevalence data used in that calculation were extracted from either Multiple Indicator Cluster Surveys (MICS) [50] or Demographic and Health Surveys (DHS) [51], both of which provide nationally representative household surveys covering several health and well-being indicators disaggregated to women and children. Between them, they cover 138 countries, providing the most comprehensive global health-related dataset currently available. The FGM/C modules used by MICS and DHS are very similar, with MICS asking 24 questions and DHS asking 21 [52,53]. Questions include knowledge of and attitudes towards the practice as well as specifics – age of cutting, type of cutter, and type of cut – of the respondents’ own FGM/C status and that of their children, thus making survey results comparable across time, country and implementing agency.

In addition to these MICS and DHS surveys, prevalence data from several other nationally representative surveys that national statistical agencies independently commissioned were included. Each of these independent surveys was, however, based on the same “gold standard” methodologies developed by the MICS and DHS surveys [54]. All the surveys referenced in this paper selected samples that were nationally representative at a 95% level of confidence [55]. The error margins on the FGM/C data were calculated at between 0.2% and 1.78% across all the included surveys based on the number of women interviewed versus the total number of women in the population at the time.

The population data used in this study were extracted from the US Census Bureau’s American Community Survey (ACS), acknowledged as “the nation’s premier source of detailed … demographic characteristics for local communities” [56]. The ACS systematically gathers data from an annual sample encompassing approximately 3.5 million households nationwide. The US Census Bureau generates yearly estimates based on data collected during a calendar year and combines data from 5 consecutive years to formulate multiyear estimates [56]. ACS data is available in two formats: aggregate ACS data, which provides a series of preformatted data tables covering various topics and geographic areas; and ACS microdata, which provides redacted individual records weighted by household or person. The ACS microdata is made available by the US Census Bureau as Public Use Microdata Sample (PUMS) files, which are limited in their geographic granularity to Public Use Microdata Areas (PUMA) – a geographic area with a minimum population of 100,000 [56]. The analysis in this paper was based on the 2015–2019 ACS PUMS dataset. The study population was selected primarily based on ancestry rather than relying on place of birth as the primary inclusion factor.

### Accounting for selective migration

#### Socio-economic driven selective migration.

National survey data by MICS and DHS showed that demographic factors such as wealth, educational attainment, and urbanisation influenced the prevalence of FGM/C in countries of origin [57]. These same socio-economic factors have been shown to influence migratory agency that results in selective migration patterns [15,17,21]. Ortensi *et al.* [19] suggested correcting for this socio-economic driven selective migration by adjusting prevalence to account for these factors based on the latest DHS or MICS survey data. Ortensi calculated a selection hypothesis coefficient based on the difference between national average prevalence rates and those for more educated, wealthier, and urban cohorts using the equation below:


$$\begin{array}{c} s_{n}=\left(\frac{\text{Pu}}{\text{Pn}}+\frac{\text{Pe}}{\text{Pn}}+\frac{\text{Pw}}{\text{Pn}}\right)/\text{3} \end{array}$$

Where:

Pu = Prevalence in the urban population

Pe = Prevalence in the more educated population-

Pw = Prevalence in the wealthier population

Pn = National average prevalence

$s_{n}$ = *Selection hypothesis coefficient for a specific country (n)*

### Ethnic-driven selective migration

These socio-economic influences, however, pale in comparison to the ethnic-specific nature of the practice [58,59]. To adjust for the impact of ethnic- and religious-based selective migration, an additional factor was introduced to the extrapolation equation. The *Ethno-religious Loading Factor* (ELF) was used to assess the potential impact of ethnic and religious selection in two populations. A sensitivity analysis was conducted, initially assuming no impact resulting from ethnic and religious selection (ELF = 1). This was followed by a scenario where ethnic and religious selection was considered to have an impact. In that scenario, the factor was set to double (ELF = 2) the age-disaggregated prevalence in the Nigerian population to account for the doubling effect of selective migration evident from previous studies. Similarly, to account for religiously driven selective migration in the Egyptian population, the factor was set to halve the risk to Egyptian girls growing up in the diaspora (ELF = 0.5). In the Nigerian case, ethnic and geographic selective migration likely impacted both the women and girls migrating with FGM/C and the risk to Nigerian girls growing up in the US. On the other hand, the prevalence of FGM/C in Egypt does not differentiate by religion [30]; however, in the migratory context, unsupportive attitudes likely result in a drop in risk for Egyptian girls growing up in the US. These ELF settings are speculative and are included to demonstrate the potential impact of ethnic and religious selective migration. The final results are presented in two forms, first with the ELF and then without the ELF to clearly demonstrate the impact.

### Accounting for migration timing

Migration timing and its interplay with the age of FGM/C determine an individual’s FGM/C status at the time of migration. Kawous *et al.* [10] proposed accounting for this by segmenting the study population into five sub-populations based on nativity, age of migration, current age and assumptions regarding FGM/C risk boundaries. In so doing Kawous *et al.* [10] effectively added five new variables to the extrapolation formula: the probability of FGM/C prior to migration (PM), since migration (SM) and the probability of future risk (FR) of FGM/C. They also segmented the impacted population to calculate the number of individuals who were living with FGM/C (LW) and those who were at risk of FGM/C (AR). This paper explores three methods by which to estimate the FGM/C risk boundaries used in these calculations.

### Method 1: Replicating Kawous et al.’s modelling

The FGM/C risk boundaries used in Kawous *et al.’s* [10] modelling were based on the assumption that those over the age of 19 were no longer at risk of FGM/C. To operationalise that assumption, segmentation of the study population extracted from the ACS 2015–2019 dataset was based on readily available data points.

### Method 2: Considering the age of FGM/C

Building on the work of Kawous *et al.* [10] and the EIGE [9], the study population was again segmented into five sub-populations, but this time, rather than assuming that the FGM/C risk boundary was the same for all ethnicities, the modelling considered the age of FGM/C in each country of origin [5]. The 25^th^, 50^th^ and 75^th^ percentile ages of FGM/C for 15 countries were extracted from UNFPA data [5]. The others were obtained from the relevant survey data. For the two countries (Nigeria and Yemen) that had a 75^th^ percentile of 0, the 25^th^ percentile was set to 0.25, the 50^th^ percentile to 0.5, and the 75^th^ percentile to 0.75, in order to capture children between the ages of 0 and 1 in the calculations. Based on the interquartile range (IQR), the upper and lower bounds for outliers were calculated based on Tukey’s method [60]. The lower fence (q1−1.5×IQR) was truncated at birth, while the upper fence (q3+1.5×IQR) was truncated at 18, so that the risk was focused on children. In this method, the universal risk boundary, which Kawous *et al*. [10] set at age 19, was replaced with the age above which FGM/C is rare in each community (q3+1.5×IQR).

### Method 3: Considering the distribution of FGM/C beyond a single fixed point

The risk boundaries in Model 1 were set at 19, while in Model 2 they were set to the age above which FGM/C is rare (q3+1.5 ×IQR). However, it will be shown that in both cases these settings likely inflated the number of girls at risk and underestimated the number already living with FGM/C. To address this challenge, a generalised gamma distribution was used to estimate the three migration timing variables: the proportion of risk prior to migration (PM), the proportion of risk since migration (SM) and the proportion of risk remaining in the future (FR). A gamma distribution provides a continuous probability distribution for a positive-valued variable and is used to model wait times until events occur. It is defined by two parameters, one that determines the curve’s shape, and the other that determines its scale [61]. Stacey [62] developed a generalised form of the gamma distribution parameterised by shape (d), power (p), and scale (a). The generalised gamma distribution provides a flexible solution to describe the diversity in age of FGM/C across the study population, because several well-known distributions appear as special cases under particular combinations of the parameters, including a gamma distribution when p=1, approximating a normal distribution in cases where d/p is large and p is small, and an exponential distribution when p=d=1. However, when d<1 the generalised gamma distribution peaks sharply on the left creating an L-shaped curve. That flexibility enabled a standard set of parameters (d, p, a) to describe the proportional distribution of risk of FGM/C across the age of FGM/C window for all populations of interest. Rather than using a simpler alternative, such as an empirical cumulative distribution based on MICS and DHS age categories, fitting the generalised gamma distribution provides a continuous, statistically smoothed probability curve that enables precise estimation at the exact age milestones associated with migration history.

The Stacy parameters (d, p, a) were estimated from the quartiles in S6 Table using a three-stage process in R version 4.2.2 [63]. First, an analytical root-finding approach solved for a gamma shape that reproduced the observed interquartile ratios exactly using the uniroot function in R (n = 10). If that approach failed, an attempt was made to identify a gamma curve with a near-exact fit (a maximum relative error of < 10% across all quartiles) using a dense numeric search of 800 parameter combinations that minimised the quartile ratio error (n = 5). The remaining cases (n = 6) were fitted to a gamma curve by bounded nonlinear optimisation using the R function nlminb. To prevent the optimiser from getting stuck in local minima, it was initialised with five diverse starting parameters. The objective function minimised the weighted sum of squared log-relative errors between the fitted quartiles and the observed quartiles, with a 50% higher weight applied to the median to prioritise central accuracy. A lower bound of 10^–12^ was used within the log-space transformations to prevent log(0) errors in cases where the input data approached zero (Nigeria, Yemen, Eritrea, Ethiopia, Indonesia, and Senegal).

By making the choice to fit generalised gamma distributions to the age of FGM/C data, it was assumed that each distribution was unimodal. That assumption was based on an analysis of the interquartile range (IQR) of each population calculated from the quartile data. That analysis shows that 66.7% (n = 14) had an IQR of 5 years or less, suggesting a unimodal distribution. The remaining seven – Ethiopia, Ghana, Kenya, Liberia, Tanzania, Togo and Uganda – had an IQR of between 6 and 10 years, potentially pointing towards a multimodal distribution in those populations. The impact of four of those was minimal, as they represented an insignificant proportion of the impacted population: Ghana (0.25%), Tanzania (0.1%), Togo (0.04%) and Uganda (0.02%). With a slightly larger impacted population, Kenya (1.73%) and Liberia (2.76%) warranted closer inspection, while the scale of Ethiopia’s population (15.53%) potentially presented a significant issue. In Kenya, the latest DHS data pointed to a unimodal distribution with 2% cut before 5 years of age, 30% between ages 5 and 9, 45% between the ages of 10 and 14 and 21% after the age of 14 [64]. Likewise, in Ethiopia, the DHS data pointed to a unimodal distribution, with 49% of FGM/C occurring below age 5, 22% between the ages of 5 and 9, 18% between the ages of 10 and 14, and 6% at age 15 or older [65]. In Liberia, on the other hand, the latest DHS survey found that 25% of FGM/C occurred before the age of 5, 17% between the ages of 5 and 9, 33% between the ages of 10 and 14 and 22% from age 15 onwards [66], suggesting a bimodal distribution with peaks in the 0–4 and 10–14 age ranges. While it is clear that any modelling introduces error through less-than-perfect fit, in this case, most of that impact was observed in smoothing out bumps in the tails of the distribution and was thus judged to be minimal.

### Accounting for structural constraints

Migration exposes individuals to new constraints, such as legislation and law enforcement that were not present in their countries of origin. Kawous *et al.* [10] accounted for this impact through the introduction of a *Prevention Impact Factor* (PIF), which they applied at various levels – 0%, 25%, 50% and 75% – to create varying risk profiles. While Kawous *et al.* [10] applied these settings without much theoretical backing, the criminology literature offers a framework by which to inform policy responses based on sensitivity analysis of the PIF settings. Both the deterrent and the labelling approaches would lead to a lower *Prevention Impact Factor* setting, while policies that achieve a desistance approach would likely result in a greater reduction of risk and thus set the *Prevention Impact Factor* higher.

While the *Prevention Impact Factor* would not apply to those cut prior to migration, its effects would equally apply to native-born and foreign-born individuals who are potentially at risk in the destination country. In this modelling, the *Prevention Impact Factor* settings were aligned with the criminology theory to create hypothetical scenarios as follows: Labelling (PIF = 0); deterrence (PIF = 0.25); desistance (PIF = 0.5). These three theories operate within an independent decision-making context; however, since decision-making regarding FGM/C operates as an interdependent process, it would hypothetically take community-led collective abandonment to achieve a higher *Prevention Impact Factor* (PIF = 0.75).

### A new extrapolation formula

To account for the impact of migration on the prevalence and risk of FGM/C in immigrant and diaspora populations, the extrapolation formula was updated as follows:

Those living with FGM/C were estimated using the following calculation:


$$\begin{array}{c} \text{LW}=\sum_{\text{n}=\text{1}}^{\text{c}}\left({\text{Pr}}_{(\text{x},\text{y}),\text{n}}\;\times \;{\text{p}}_{(\text{x},\text{y}),\text{n}}\;\times \;{\text{s}}_{\text{n}}\times \;{\text{ELF}}_{\text{nav},\text{n}}\times \text{PM}\right)\; \end{array}$$

$$\begin{array}{c} +\sum_{\text{n}=\text{1}}^{\text{c}}\left({\text{Pr}}_{(\text{x},\text{y}),\text{n}}\;\times \;{\text{p}}_{(\text{x},\text{y}),\text{n}}\;\times \;{\text{s}}_{\text{n}}\times {\text{ELF}}_{\text{nav},\text{n}}\times \text{SM}\times (\text{1}-\text{PIF})\right)\; \end{array}$$

While those at risk of FGM/C were estimated using the following calculation:


$$\begin{array}{c} \text{AR}=\sum_{\text{n}=\text{1}}^{\text{c}}\left({\text{Pr}}_{(\text{x},\text{y}),\text{n}}\;\times \;{\text{p}}_{(\text{x},\text{y}),\text{n}}\;\times \;{\text{s}}_{\text{n}}\times {\text{ELF}}_{\text{nav},\text{n}}\times \text{FR}\times (\text{1}-\text{PIF})\right)\; \end{array}$$

The sum of those two results produced the impacted population.

Where:

${\text{Pr}}_{(\text{x},\text{y}),\text{n}}$ = the FGM/C prevalence segmented by five-year age cohorts (x,y) in a specific country-of-origin (n)

${\text{p}}_{(\text{x},\text{y}),\text{n}}\;$ = the study population in the country-of-residence associated with a specific country-of-origin (n) segmented by five-year age cohorts (x,y)

(x,y) = five-year age cohorts from 0 to 4, 5–9, etc up to 75–79 and 80+

${\text{s}}_{\text{n}}$ = the *selection hypothesis coefficient* in a specific country-of-origin (n)

${\text{ELF}}_{\text{nav},\text{n}}$ = the *Ethno-religious Loading Factor* disaggregated by nativity in a specific country-of-origin (n)

PM = Proportion of risk prior to migration

SM = Proportion of risk since migration

FR = Proportion of future risk

PIF = *Prevention Impact Factor* set to 0, 0.25, 0.50, and 0.75

c = the number of countries of origin under investigation

## Results

### Data sources

The age-disaggregated prevalence used in this study (S1 Table) was calculated based on the methodology developed by Callaghan [49]. The age-disaggregated population used in this study are shown in S2 Table. The study population consisted of 1,248,776 women and girls associated with 21 countries of origin. The 15- to 49-year-old national survey data used to calculate age-disaggregated prevalence are shown in S3 Table, while the latest 15- to 49-year-old national survey data, together with the national average prevalence used in Jones *et al.* [13] and Goldberg *et al.* [14] method estimations and the *selection hypothesis coefficients* are shown in S4 Table.

### Baseline results based on previous US and European methods

While previous studies in the US did not account for the impact of migration and acculturation, more recent European studies did. Following the US methods of Jones *et al.* [13] and Goldberg *et al.* [14], the impacted population in the US in 2019 was estimated at 620,595 and 577,166, respectively. Likewise, applying the basic extrapolation method, which also did not account for the impact of migration and acculturation, using disaggregated prevalence and study population (both first- and second-generation) data, the impacted population was estimated at 586,245. On the other hand, following a process similar to Stegner et al. [12], in which native-born girls were considered to be at half the risk (m = 0.5), the impacted population was estimated at 505,395.

Extending Stegner et al.’s [12] method by extrapolating prevalence based on the method previously developed by Callaghan [49] (S1 Table), thereby aligning the prevalence and population cohorts and accounting for social desirability bias, reduced the high road estimate (m = 0) to 572,569, and the low road estimate (m = 0.5) to 495,996.

### Selective migration

Building on these baseline estimates, to account for socio-economic selective migration by including Ortensi *et al*.’s [19] *selection hypothesis coefficient*, further reduced the high road estimate to 542,225, and the low road estimate to 467,801. Accounting for ethno-religious selective migration, through the inclusion of the hypothetical *Ethno-religious Loading Factor* (ELF), increased the high road estimate to 548,243, and the low road estimate to 477,816.

### Migration timing

Migration timing was modelled using three methods

**Method 1:** In the first method, the *Age of FGM/C Risk Boundaries* were set to 19 across the entire study population. The results segmented the study population by nativity, age of migration, the age of FGM/C risk boundaries, and current age, based on Kawous *et al.*’s [10] method.

**Method 2:** In the second method, the *Age of FGM/C Risk Boundaries* (q3+1.5 ×IQR) were calculated for each population as shown in S5 Table. As can be seen from the boxplots of the *Age of FGM/C Risk Boundaries* (Fig 1) while a Ugandan girl is at risk throughout her childhood as assumed by Kawous *et al.* [10], a 5-year-old Yemeni child is no longer at risk as she has already passed the age of FGM/C in her community, while a 5-year-old Egyptian child has not yet entered the FGM/C risk window, and so all of her risk remains in the future.

**Fig 1:**
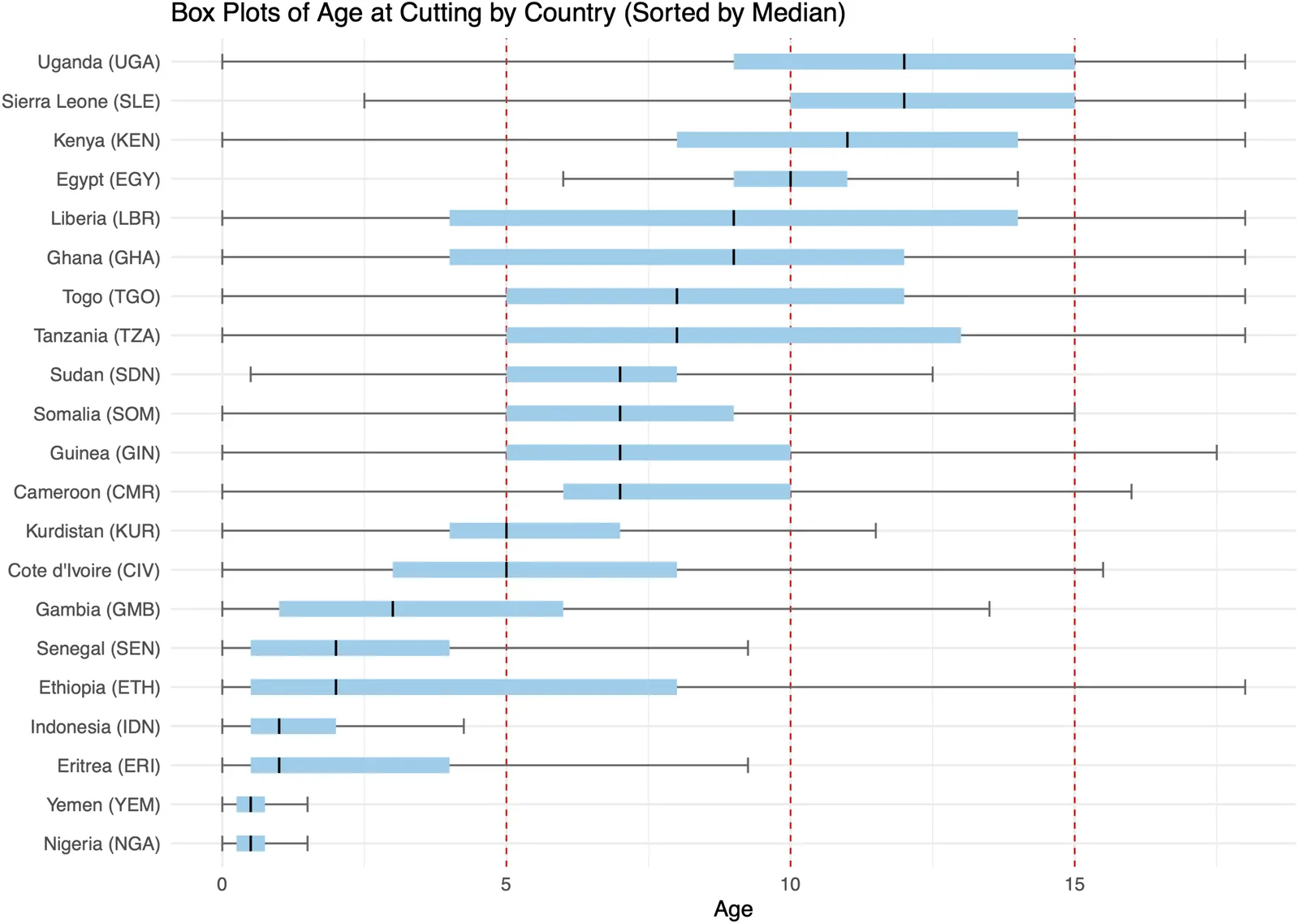
Boxplots created in R to illustrate the distribution of the Age of FGM/C Risk Boundaries sorted by median age.

**Method 3**: The third method estimated the distribution of FGM/C risk across age ranges by fitting a generalised gamma distribution to each ethnicity. The results, as shown in S6 Table, show that 95.2% of the fitted quartiles fell within the 95% confidence level of the original quartiles, and all fell within the 90% confidence level. Turning to the case of Liberia, the fitted quartiles were all within the 99% confidence level of the original quartiles, suggesting that the gamma distribution was able to match the observed distribution despite the potential bimodal nature of the practice

The resultant probability distributions were used to estimate the proportion of FGM/C risk relative to two events: the age of migration and the current age (in 2019) of each record in the study population. In the case of immigrants, these results were used to calculate the proportion of risk of FGM/C that occurred prior to migration (*PM*), the proportion of risk since migration (*SM*), and the proportion of risk that remained (*FR*). For those born in the US, the distribution was split at the individual’s current age to establish the proportion of historic risk (*SM*) versus the proportion of risk that remained (*FR*).

Taking Egypt as an example, the 25^th^ quartile was 9, the 50^th^ 10 and the 75^th^ 11 years of age [5]. A generalised gamma was identified that exactly fitted the data with parameters d=15.760, p=3.041 and a=5.949. The PDF (Fig 2) suggests that the age below which FGM/C rarely occurs was five, while the age above which FGM/C rarely occurs was 15. Taking the example of an Egyptian girl who migrated at 8 and was aged 12 in 2019 (the year of the study population), three proportional risks were calculated: The proportional risk of FGM/C prior to migration (*PM*) was calculated as the area below the curve to the left of the age of migration line (shaded in blue in Fig 2); the proportional risk of FGM/C since migration (*SM*) was calculated as the area below the curve between the age of migration and the current age in 2019 (shaded in green in Fig 2); and the proportional future risk (*FR*) of FGM/C was calculated as the area below the curve to the right of the age in 2019 (shaded in red in Fig 2). These three proportional risk estimates were calculated for each record in the study population.

**Fig 2:**
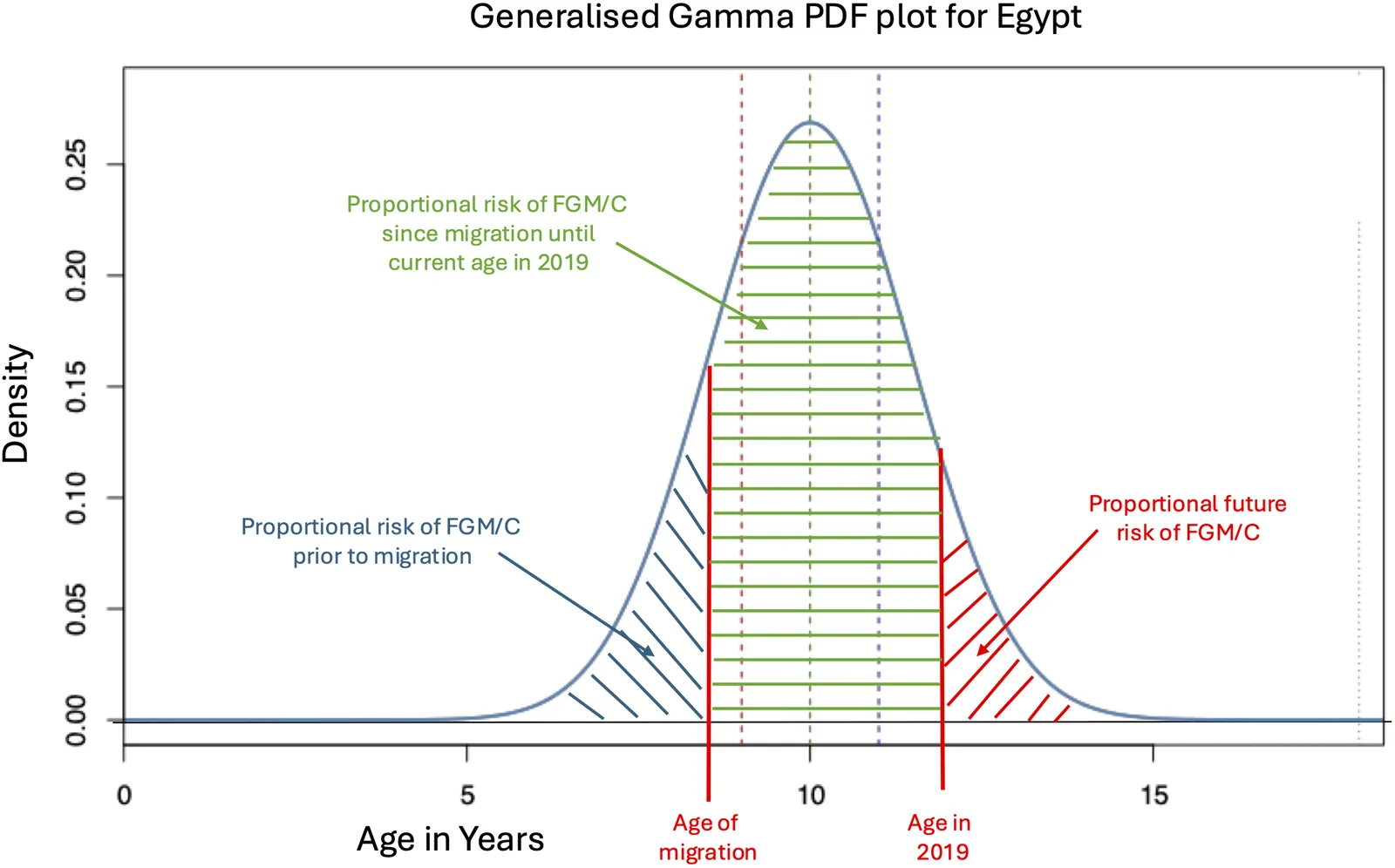
Proportional distribution of FGM/C graph for the Egyptian population showing an imaginary age of migration and current age for purposes of demonstration.

### Comparison of results calculated using the three methods

Each of the methods described above was used to estimate the number of individuals in each subgroup, as shown in Table 1. In Method 1, the risk boundary was set at 19, in Method 2, it was set at (q3+1.5*IQR), while in Method 3, the calculations were based on the generalised gamma proportional risk estimates. The results presented in Table 1 are based on calculations using the age-disaggregated population and prevalence data together with the *selection hypothesis coefficient,* the *Ethno-religious Loading Factor* and the applicable migration timing method.

**Table 1:** The estimated Impacted Population segmented into several sub-groups based on nativity and migration timing. Showing the estimates for all three methods.

Population Segment	Method 1	Method 2	Method 3
1a: Foreign-born and cut before migration	311,311	365,263	400,954
1b: Foreign-born and cut after migration	92,883	58,291	37,445
1c: Foreign-born & potentially at risk after migration	42,338	22,977	8,133
2a: Native-born and cut	55,246	80,683	106,553
2b: Native-born and potentially at risk	85,608	60,172	34,301
**Population impacted by FGM/C (1a + 1b + 1c + 2a + 2b)**	587,386	587,386	587,386
Living with FGM/C (1a + 1b + 2a)	459,440	504,237	544,952
At risk of FGM/C (1c + 2b)	127,946	83,149	42,434
Estimate of FGM/C that occurred while US-based (1b + 2a)	148,129	138,974	143,998
Study population (SP)	1,248,776	1,248,776	1,248,776
**Population not impacted by FGM/C (SP – Impacted)**	661,390	661,390	661,390

Comparing the results, as shown in Table 1, clearly shows the merit of the more nuanced generalised gamma distribution approach. While all three approaches estimated that FGM/C impacted 587,386 women and girls, Methods 2 and 3 both increased the estimate of those who were cut prior to migration and reduced the estimates of those who were either cut after migration or remained at risk. Likewise, the number of native-born women and girls who were already cut increased while the number of native-born girls at risk was reduced by the same amount. In so doing, the more nuanced approach reclassified 85,512 women and girls as living with, rather than at risk of, FGM/C. This represented a 66.8% drop in the number of girls thought to be at risk. That shift in individuals from the at-risk to the living-with categories has significant policy impacts.

### Structural constraints

The results of a sensitivity analysis of the different *Prevention Impact Factor* values are shown in Table 2. Since most FGM/C occurs prior to migration, the *Prevention Impact Factor* has a limited impact on the estimation of those living with FGM/C, but a much larger impact on the at-risk estimate.

**Table 2:** Results based on the generalised gamma distribution results shown in S6 Table, the Prevention Impact Factor was set to 0, 0.25, 0.50, and 0.75.

Population Segment	Labelling PIF = 0	Deterrence PIF = 0.25	Desistance PIF = 0.50	Community abandon-ment PIF = 0.75
1a: Foreign-born and cut before migration	400,954	400,954	400,954	400,954
1b: Foreign-born and cut after migration	37,445	28,084	18,723	9,361
1c: Foreign-born & potentially at risk after migration	8,133	6,100	4,067	2,033
2a: Native-born and cut	106,553	79,915	53,277	26,638
2b: Native-born and potentially at risk	34,301	25,726	17,151	8,575
**Population impacted by FGM/C (1a + 1b + 1c + 2a + 2b)**	**587,386**	**540,778**	**494,170**	**447,562**
Living with FGM/C (1a + 1b + 2a)	544,952	508,953	472,953	436,954
At risk of FGM/C (1c + 2b)	42,434	31,826	21,217	10,609
Estimate of FGM/C that occurred while US-based (1b + 2a)	143,998	107,999	71,999	36,000
Study population (SP)	1,248,776	1,248,776	1,248,776	1,248,776
**Population not impacted by FGM/C (SP – Impacted)**	**661,390**	**707,998**	**754,606**	**801,214**

### The impact of the ELF

To assess the impact of the *Ethno-religious Loading Factor* (ELF), Table 3 presents the same results as Table 2, but removes its effects (ELF = 1) from all populations. Removing the effects of the ELF from the results reduces the living-with population by between 9.7% and 12.1% depending on the PIF setting, while it increases the at-risk population by 17.8% across all PIF scenarios.

**Table 3:** Results based on the generalised gamma distribution results shown in S6 Table, with the ELF = 1 for all populations and the Prevention Impact Factor was set to 0, 0.25, 0.50, and 0.75.

Population Segment	Labelling PIF = 0	Deterrence PIF = 0.25	Desistance PIF = 0.50	Community abandon-ment PIF = 0.75
1a: Foreign-born and cut before migration	348,231	348,231	348,231	348,231
1b: Foreign-born and cut after migration	36,995	27,746	18,498	9,249
1c: Foreign-born & potentially at risk after migration	8,122	6,092	4,061	2,031
2a: Native-born and cut	107,025	80,269	53,513	26,756
2b: Native-born and potentially at risk	41,883	31,412	20,942	10,471
**Population impacted by FGM/C (1a + 1b + 1c + 2a + 2b)**	**542,256**	**493,750**	**445,244**	**396,737**
Living with FGM/C (1a + 1b + 2a)	492,251	456,246	420,241	384,236
At risk of FGM/C (1c + 2b)	50,005	37,504	25,003	12,501
Estimate of FGM/C that occurred while US-based (1b + 2a)	144,020	108,015	72,010	36,005
Study population (SP)	1,248,776	1,248,777	1,248,778	1,248,779
**Population not impacted by FGM/C (SP – Impacted)**	**706,520**	**755,027**	**803,535**	**852,042**

## Discussion

Previous estimates of FGM/C in the US and Europe likely overestimated the impacted population because they did not accurately account for the profound impact of migration. The modelling developed in this paper attempted to quantify that impact and, in so doing, demonstrated that Jones et al. [13] and Goldberg *et al.* [14] likely overestimated the impacted population by 12.9% (n = 79,817) and 6.3% (n = 36,388), respectively, when setting the PIF to represent the hypothesised deterrence scenario (PIF = 0.25) and considering the hypothesised impact of ethno-religious selective migration. Removing the ELF from the estimation suggests that Jones et al. [13] and Goldberg *et al.* [14] likely overestimated the impacted population by 20.4% (n = 126,845) and 14.5% (n = 83,416), respectively. Furthermore, Goldberg *et al.* reported their results as the number of women and girls who “were at risk of FGM/C or its consequences” [14], which led many to interpret their data as “500,000 women and girls in the United States are at risk of genital mutilation” [67]. The modelling in this paper suggests that this characterisation of the data could not be further from the truth. Segmenting the impacted population into those who are likely living with FGM/C and those who potentially remain at risk showed that an overwhelming 94.1% (n = 508,953) were likely already living with FGM/C, while only 5.9% (n = 31,826) were thought to be at risk. The primary driver of that shift from at-risk to living-with was modelling the distribution of ages at which FGM/C occurs in each ethnic community. The modelling also suggests that while the risk of cutting is overwhelmingly confined to childhood in most populations (F(18) ≥ 0.95 in 15 of the 21 populations in the generalised gamma distributions shown in S6 Table), it also suggests that risk potentially extends into adulthood in some communities – notably Liberia, Ethiopia and Tanzania.

There are obvious policy implications that result from the potential misrepresentation of the impacted population inherent in previous studies. These implications ripple through the entire response strategy. Not only do they potentially impede policy formulation [68], but they also likely frustrate partnership development with impacted communities, who often feel labelled by misguided policy responses [45]. The resultant vilification of impacted communities [46] potentially undermines the other four responses – prevention, protection, provision and prosecution – outlined by Mergaert *et al.* [48] in their 7P framework. In reality, the reclassification from at risk to living with FGM/C does not reduce the overall prevalence – girls are still being cut – it simply displaces the cutting to the period prior to migration. This finding alone has massive policy implications – the need for child protection is likely less than previously thought, and much more attention needs to be focused on the provision of services to survivors.

It is clear that addressing FGM/C in diaspora contexts requires the establishment and strengthening of partnerships between several stakeholders, including affected communities, civil society organisations, frontline service providers, and local, county, state and federal governments. While many of these partnerships already exist, it is critical that they are strengthened to support affected women and girls. Adopting a comprehensive approach to FGM/C that brings together prevalence, partnership, policy, prevention, protection, provision, and prosecution is vital to efforts to support the communities affected by this practice. Emphasis should be placed on community-led efforts at prevention and strengthening the provision of services to survivors. Child protection and prosecution efforts should be seen as the last lines of defence rather than fronted as a primary response to the practice so as to avoid reinforcing a culture of silence and the labelling and stereotyping of impacted communities.

### Limitations

Four primary limitations are identified. The values for the *Ethno-religious Loading Factor* (ELF) for the Egyptian and Nigerian populations are speculative and included to demonstrate the potential impact rather than predict the exact scale of the mechanism. The generalised gamma distribution modelling assumes unimodal age distributions of FGM/C. The impact of long-term residence in refugee camps prior to migration has not been accounted for in the modelling. Furthermore, this paper has not modelled the impact of transnational travel or acculturation – each of which requires additional research to effectively quantify. However, the expanded extrapolation formula presented below provides the mechanism by which to include these effects in the calculation.

### Future research

#### Accounting for transnational travel.

While it is clear that transnational travel poses a risk to both native- and foreign-born girls during visits to their parents’ countries of origin [33,45,69–73], the extent of these risks is difficult to quantify. Notwithstanding the challenge of quantifying the scale, it is possible to establish the modelling framework. From a modelling perspective, a *Transnational Travel Risk Factor* (TTRF) could be introduced. The TTRF would be an estimate of the proportion of girls who were exposed to the risks associated with transnational travel at the ethnic community level. Setting the *Transnational Travel Risk Factor* to 0 effectively removes its influence from the equation, while setting it to 1 removes the protective impact of the *Prevention Impact Factor (PIF)*. A setting between those two extremes removes the protective impact from the subset of girls who are exposed to risk in countries of origin as a result of transnational travel.

#### Accounting for acculturation.

The specifics of migration also influence individuals’ acculturation strategies and experiences. On the one hand, migration potentially provides a catalyst for attitude change through exposure to new knowledge, affecting acculturation responses [31–35]. On the other hand, migration timing determines the extent of exposure an individual has to the destination context [31,38,40–42,74]. Moreover, migration networks and destination locations affect the extent to which individuals are located in closed communities, which, combined with migration aspirations, suggest different acculturation strategies and experiences [15,36,45,75,76]. The effects of acculturation, once they are estimated, could be modelled using the *Acculturation Impact Factor* (AIF).

### An expanded extrapolation formula

Bringing these additions together, the expanded extrapolation formula could be presented as follows:

Those living with FGM/C could be estimated using the following calculation:


$$\begin{array}{c} \text{LW}=\sum_{\text{n}=\text{1}}^{\text{c}}\left({\text{Pr}}_{(\text{x},\text{y}),\text{n}}\;\times \;{\text{p}}_{(\text{x},\text{y}),\text{n}}\;\times \;{\text{s}}_{\text{n}}\times \;{\text{ELF}}_{\text{nav},\text{n}}\times \text{PM}\right)\; \end{array}$$

$$\begin{array}{c} +\sum_{\text{n}=\text{1}}^{\text{c}}\left({\text{Pr}}_{(\text{x},\text{y}),\text{n}}\;\times \;{\text{p}}_{(\text{x},\text{y}),\text{n}}\;\times \;{\text{s}}_{\text{n}}\times {\text{ELF}}_{\text{nav},\text{n}}\times \text{SM}\times (\left(\left(\text{1}-{\text{TTRF}}_{\text{n}}\right)\times (\text{1}-\text{PIF})\right)+\;{\text{TTRF}}_{\text{n}})\times (\text{1}-\text{AI})\right)\; \end{array}$$

While those at risk of FGM/C could be estimated using the following calculation:


$$\begin{array}{c} \text{AR}=\sum_{\text{n}=\text{1}}^{\text{c}}\left({\text{Pr}}_{(\text{x},\text{y}),\text{n}}\;\times \;{\text{p}}_{(\text{x},\text{y}),\text{n}}\;\times \;{\text{s}}_{\text{n}}\times {\text{ELF}}_{\text{nav},\text{n}}\times \text{FR }\times (\left(\left(\text{1}-{\text{TTRF}}_{\text{n}}\right)\times (\text{1}-\text{PIF})\right)+{\text{TTRF}}_{\text{n}})\times (\text{1}-\text{AI})\right)\; \end{array}$$

The sum of those two results would produce the impacted population.

Where:

${\text{Pr}}_{(\text{x},\text{y}),\text{n}}$ = the FGM/C prevalence segmented by five-year age cohorts (x,y) in a specific country-of-origin (n)

${\text{p}}_{(\text{x},\text{y}),\text{n}}\;$ = the study population in the country-of-residence associated with a specific country-of-origin (n) segmented by five-year age cohorts (x,y)

(x,y) = five-year age cohorts from 0 to 4, 5–9, etc up to 75–79 and 80+

${\text{s}}_{\text{n}}$ = the *Selection Hypothesis coefficient* in a specific country-of-origin (n)

${\text{ELF}}_{\text{nav},\text{n}}$ = the *Ethno-religious Loading Factor* disaggregated by nativity in a specific country-of-origin (n)

PM = Proportion of risk prior to migration

SM = Proportion of risk since migration

FR = Proportion of future risk

PIF = *Prevention Impact Factor* set to 0, 0.25, 0.50, and 0.75

TTRF_n_ = the *Transnational Travel Risk Factor* set between 0 and 1 to represent the proportion of the population for a specific ethnic community (n) who were exposed to risk through transnational travel.

AI = *Acculturation Impact Factor* set between 0 and 1 to represent the effects of acculturation

c = the number of countries of origin under investigation

### Declaration AI-Assisted Technologies

The author used Gemini (accessed between October 2025 and June 2026) to assist with writing the R code used to fit the generalised gamma distributions to the age of FGM/C data. The author subsequently reviewed the code, methodology, and results and takes full responsibility for the manuscript’s content. The resultant R code is available in protocols.io [63].

## Supporting information

S1 Table Calculated age-disaggregated prevalence of FGM/C for 21 countries, calculated based on the methodology developed by Callaghan [49] from nationally representative survey data. (DOCX)

S2 Table Study population (SP) selected out of ACS 2015−19 and assigned an ethnicity based primarily on ancestry rather than place of birth. (DOCX)

S3 Table FGM/C prevalence extracted from 82 nationally representative surveys sorted by country and year of survey. (DOCX)

S4 Table FGM/C prevalence from the latest nationally representative survey showing age cohort and national prevalence data, together with the prevalence by urban, education and wealth to calculate the Migration Selection Factor. (DOCX)

S5 Table Age of FGM/C Risk Boundaries. (DOCX)

S6 Table The results of the generalised gamma fitting showing the original observed quartiles, the method used to estimate each result, the fitted quartiles, the margin of error between the observed and fitted quartiles, the Stacy generalised gamma parameters d, p and a, and the cumulative proportion of the distribution at age 18. (DOCX)
