# Supplementary material for: Modelling the impact of migration on FGM/C prevalence and risk in immigrant and diaspora populations
Source: PLoS One. 2026 Oct 13;21(10):e0360038. doi: 10.1371/journal.pone.0360038 (PMC13660914; doi:10.1371/journal.pone.0360038)
Supplement: Supplementary file 1 [file pone.0360038.s001.docx]

Supplemental Data Table 1: Calculated age-disaggregated prevalence of FGM/C for 21 countries, calculated based on the methodology developed by Callaghan [56] from nationally representative survey data

| **Country / Ethnicity** | **00-04** | **05-09** | **10-14** | **15-19** | **20-24** | **25-29** | **30-34** | **35-39** | **40-44** | **45-49** | **50-54** | **55-59** | **60-64** | **65-69** | **70-74** | **75-79** | **80+** |
| --- | --- | --- | --- | --- | --- | --- | --- | --- | --- | --- | --- | --- | --- | --- | --- | --- | --- |
| Cameroon | 0.00 | 0.00 | 0.00 | 0.00 | 0.01 | 0.06 | 0.40 | 2.50 | 1.60 | 1.10 | 1.20 | 1.80 | 2.40 | 2.40 | 2.40 | 2.40 | 2.40 |
| Cote d'Ivoire | 23.65 | 25.19 | 27.01 | 29.17 | 31.72 | 34.66 | 37.13 | 39.01 | 41.78 | 43.16 | 44.73 | 43.66 | 45.68 | 45.60 | 45.60 | 45.60 | 45.60 |
| Egypt | 82.94 | 84.25 | 85.77 | 87.55 | 89.62 | 91.83 | 93.88 | 95.57 | 96.17 | 96.33 | 96.33 | 96.28 | 96.57 | 96.77 | 96.73 | 96.73 | 96.73 |
| Eritrea | 45.73 | 49.97 | 54.70 | 59.99 | 65.92 | 72.55 | 79.59 | 85.67 | 89.88 | 92.59 | 93.66 | 94.16 | 94.84 | 94.75 | 95.07 | 95.07 | 95.07 |
| Ethiopia | 27.09 | 31.54 | 36.80 | 43.04 | 50.46 | 58.59 | 66.94 | 74.04 | 76.80 | 78.67 | 81.86 | 81.39 | 81.83 | 82.10 | 82.10 | 82.10 | 82.10 |
| Gambia | 79.82 | 79.06 | 78.33 | 77.64 | 76.98 | 76.83 | 75.34 | 75.17 | 75.01 | 75.27 | 75.68 | 75.82 | 75.40 | 75.40 | 75.40 | 75.40 | 75.40 |
| Ghana | 0.54 | 0.65 | 0.80 | 1.04 | 1.44 | 1.81 | 2.54 | 3.19 | 4.53 | 5.65 | 5.87 | 5.77 | 6.53 | 6.65 | 6.65 | 6.65 | 6.65 |
| Guinea | 81.92 | 84.04 | 86.24 | 88.52 | 90.89 | 92.89 | 94.77 | 96.45 | 97.58 | 98.39 | 98.66 | 98.70 | 98.96 | 99.00 | 99.00 | 99.00 | 99.00 |
| Indonesia | 49.00 | 49.00 | 49.00 | 49.00 | 49.00 | 49.00 | 49.00 | 49.00 | 49.00 | 49.00 | 49.00 | 49.00 | 49.00 | 49.00 | 49.00 | 49.00 | 49.00 |
| Kenya | 4.48 | 5.70 | 7.26 | 9.30 | 11.94 | 15.40 | 19.71 | 24.27 | 30.03 | 35.76 | 40.22 | 45.82 | 46.29 | 46.22 | 46.23 | 46.23 | 46.23 |
| Kurdistan | 3.30 | 5.59 | 9.59 | 16.66 | 29.30 | 34.22 | 48.44 | 54.29 | 60.95 | 56.23 | 54.99 | 56.40 | 56.40 | 56.40 | 56.40 | 56.40 | 56.40 |
| Liberia | 22.02 | 22.56 | 23.59 | 25.24 | 27.65 | 35.34 | 45.41 | 55.64 | 56.49 | 61.93 | 65.14 | 66.05 | 66.05 | 66.05 | 66.05 | 66.05 | 66.05 |
| Nigeria | 6.92 | 8.28 | 9.95 | 12.02 | 14.60 | 17.59 | 20.42 | 23.49 | 26.72 | 29.09 | 31.16 | 32.72 | 33.99 | 34.17 | 34.17 | 34.17 | 34.17 |
| Senegal | 18.62 | 19.39 | 20.23 | 21.14 | 22.12 | 23.68 | 25.03 | 25.71 | 25.97 | 26.51 | 26.39 | 26.50 | 26.53 | 26.53 | 26.53 | 26.53 | 26.53 |
| Sierra Leone | 30.18 | 36.97 | 45.44 | 56.02 | 69.31 | 80.50 | 89.42 | 94.18 | 95.45 | 96.29 | 96.32 | 96.41 | 96.40 | 96.40 | 96.40 | 96.40 | 96.40 |
| Somalia | 93.86 | 94.54 | 95.24 | 95.94 | 96.65 | 97.82 | 98.39 | 98.49 | 98.81 | 98.90 | 98.66 | 98.66 | 98.90 | 98.90 | 98.90 | 98.90 | 98.90 |
| Sudan | 67.60 | 70.29 | 73.09 | 76.01 | 79.05 | 82.21 | 84.71 | 86.36 | 86.58 | 89.50 | 90.31 | 89.91 | 90.15 | 89.97 | 90.09 | 90.60 | 90.60 |
| Tanzania | 1.66 | 2.20 | 2.97 | 4.07 | 5.69 | 7.75 | 10.39 | 13.12 | 15.90 | 17.96 | 19.35 | 19.95 | 20.71 | 21.19 | 21.33 | 21.33 | 21.33 |
| Togo | 0.34 | 0.45 | 0.60 | 0.82 | 1.15 | 1.67 | 2.87 | 4.19 | 5.58 | 6.89 | 7.69 | 8.03 | 7.95 | 7.95 | 7.95 | 7.95 | 7.95 |
| Uganda | 0.95 | 0.77 | 0.64 | 0.55 | 0.52 | 0.52 | 0.71 | 1.01 | 0.95 | 0.95 | 1.02 | 1.02 | 0.90 | 0.90 | 0.90 | 0.90 | 0.90 |
| Yemen | 10.84 | 11.54 | 12.32 | 13.19 | 14.17 | 15.27 | 16.19 | 18.99 | 21.81 | 21.98 | 22.46 | 23.06 | 23.65 | 23.92 | 23.90 | 23.90 | 23.90 |
