# Supplementary material for: Modelling the impact of migration on FGM/C prevalence and risk in immigrant and diaspora populations
Source: PLoS One. 2026 Oct 13;21(10):e0360038. doi: 10.1371/journal.pone.0360038 (PMC13660914; doi:10.1371/journal.pone.0360038)
Supplement: Supplementary file 2 [file pone.0360038.s002.docx]

Supplemental Data Table 2: Study population (SP) selected out of ACS 2015-19 and assigned an ethnicity based primarily on ancestry rather than place of birth.

|  | 00-04 yr | 05-09 yr | 10-14 yr | 15-19 yr | 20-24 yr | 25-29 yr | 30-34 yr | 35-39 yr | 40-44 yr | 45-49 yr | 50-54 yr | 55-59 yr | 60-64 yr | 65-69 yr | 70-74 yr | 75-79 yr | 80+ yr | Total |
| --- | --- | --- | --- | --- | --- | --- | --- | --- | --- | --- | --- | --- | --- | --- | --- | --- | --- | --- |
| Cameroon | 2,815 | 2,631 | 2,346 | 1,997 | 3,008 | 4,693 | 4,534 | 4,790 | 3,991 | 3,239 | 2,173 | 1,863 | 1,473 | 1,211 | 627 | 355 | 286 | 42,032 |
| Cote d'Ivoire | 36 | 34 | 196 | 654 | 740 | 640 | 923 | 585 | 680 | 517 | 622 | 405 | 205 | 96 | 24 | 0 | 0 | 6,357 |
| Egypt | 12,224 | 11,286 | 11,028 | 10,945 | 9,411 | 11,692 | 13,096 | 12,906 | 10,276 | 9,311 | 6,685 | 5,815 | 5,624 | 4,889 | 3,883 | 2,754 | 3,249 | 145,074 |
| Eritrea | 2,573 | 2,108 | 1,967 | 2,093 | 2,490 | 2,367 | 2,869 | 3,235 | 3,586 | 2,494 | 2,114 | 1,936 | 1,398 | 800 | 489 | 190 | 158 | 32,867 |
| Ethiopia | 13,529 | 13,912 | 11,610 | 10,886 | 9,453 | 12,170 | 18,278 | 19,211 | 14,973 | 12,259 | 8,992 | 6,637 | 4,590 | 3,548 | 1,780 | 1,072 | 1,069 | 163,969 |
| Gambia | 423 | 344 | 547 | 639 | 1,413 | 1,063 | 849 | 1,021 | 720 | 1,214 | 551 | 349 | 66 | 92 | 65 | 115 | 0 | 9,471 |
| Ghana | 7,084 | 6,245 | 6,647 | 5,007 | 7,860 | 8,869 | 10,278 | 11,187 | 11,433 | 9,721 | 6,950 | 6,239 | 4,595 | 3,246 | 1,556 | 928 | 226 | 108,071 |
| Guinea | 197 | 255 | 260 | 639 | 953 | 1,160 | 790 | 533 | 1,099 | 857 | 758 | 305 | 401 | 69 | 157 | 37 | 102 | 8,572 |
| Indonesia | 3,041 | 4,149 | 4,260 | 4,390 | 4,911 | 4,475 | 5,259 | 6,055 | 6,815 | 6,651 | 4,850 | 3,915 | 4,015 | 3,144 | 1,580 | 1,001 | 1,655 | 70,166 |
| Kenya | 3,854 | 5,239 | 3,473 | 5,546 | 6,821 | 6,404 | 8,188 | 7,526 | 8,827 | 4,736 | 3,804 | 2,916 | 2,411 | 1,285 | 670 | 410 | 161 | 72,271 |
| Kurdistan | 1,422 | 1,529 | 1,500 | 1,036 | 1,282 | 1,560 | 1,276 | 1,475 | 820 | 874 | 397 | 338 | 212 | 387 | 247 | 193 | 159 | 14,707 |
| Liberia | 3,979 | 3,821 | 4,438 | 5,050 | 5,001 | 5,217 | 6,143 | 5,821 | 4,802 | 4,295 | 4,315 | 3,833 | 2,395 | 1,431 | 1,293 | 746 | 1,082 | 63,662 |
| Nigeria | 18,424 | 17,632 | 17,480 | 18,902 | 19,893 | 22,224 | 23,461 | 24,813 | 19,705 | 18,534 | 16,318 | 14,186 | 9,861 | 6,969 | 4,353 | 2,699 | 2,480 | 257,934 |
| Senegal | 967 | 1,058 | 1,157 | 907 | 862 | 1,052 | 1,534 | 1,529 | 1,770 | 1,681 | 1,212 | 413 | 251 | 236 | 7 | 58 | 28 | 14,722 |
| Sierra Leone | 641 | 766 | 1,196 | 1,254 | 2,210 | 2,870 | 2,780 | 2,425 | 2,199 | 2,056 | 2,234 | 1,722 | 1,488 | 1,263 | 655 | 249 | 360 | 26,368 |
| Somalia | 13,324 | 11,335 | 8,849 | 7,778 | 8,243 | 8,629 | 9,844 | 7,120 | 5,246 | 4,469 | 2,690 | 2,053 | 1,507 | 999 | 1,099 | 519 | 1,097 | 94,801 |
| Sudan | 4,560 | 3,551 | 3,668 | 2,658 | 2,732 | 3,728 | 3,337 | 2,713 | 3,840 | 2,323 | 1,623 | 1,182 | 574 | 453 | 420 | 237 | 239 | 37,838 |
| Tanzania | 792 | 421 | 1,425 | 1,200 | 720 | 632 | 1,020 | 1,393 | 1,135 | 1,218 | 464 | 304 | 641 | 349 | 123 | 37 | 50 | 11,924 |
| Togo | 335 | 334 | 418 | 477 | 1,022 | 1,236 | 1,324 | 1,393 | 1,344 | 879 | 658 | 439 | 526 | 255 | 97 | 81 | 0 | 10,818 |
| Uganda | 763 | 1,350 | 1,012 | 802 | 739 | 2,069 | 2,038 | 1,650 | 1,524 | 1,049 | 872 | 557 | 429 | 391 | 268 | 101 | 107 | 15,721 |
| Yemen | 4,627 | 4,298 | 4,621 | 3,486 | 3,447 | 4,455 | 4,513 | 3,102 | 2,366 | 1,376 | 1,396 | 1,146 | 899 | 867 | 397 | 172 | 263 | 41,431 |
| **Study Pop (SP)** | **95,610** | **92,298** | **88,098** | **86,346** | **93,211** | **107,205** | **122,334** | **120,483** | **107,151** | **89,753** | **69,678** | **56,553** | **43,561** | **31,980** | **19,790** | **11,954** | **12,771** | **1,248,776** |
