# Supplementary material for: Modelling the impact of migration on FGM/C prevalence and risk in immigrant and diaspora populations
Source: PLoS One. 2026 Oct 13;21(10):e0360038. doi: 10.1371/journal.pone.0360038 (PMC13660914; doi:10.1371/journal.pone.0360038)
Supplement: Supplementary file 3 [file pone.0360038.s003.docx]

Supplemental Data Table 3: FGM/C prevalence extracted from 82 nationally representative surveys sorted by country and year of survey

| **Country** | **Survey** | **Age 15–19 (%)** | **Age 20–24 (%)** | **Age 25–30 (%)** | **Age 30 –34 (%)** | **Age 35–39 (%)** | **Age 40–44 (%)** | **Age 45–49 (%)** |
| --- | --- | --- | --- | --- | --- | --- | --- | --- |
| Cameroon | DHS 2004 | 0.4 | 2.5 | 1.6 | 1.1 | 1.2 | 1.8 | 2.4 |
| Côte d'Ivoire | DHS 1998-99 | 41.2 | 42.7 | 42.4 | 49.0 | 44.5 | 51.4 | 51.0 |
| Côte d'Ivoire | AIS 2005 | 39.4 | 38.7 | 41.9 | 42.5 | 44.7 | 44.1 | 48.9 |
| Côte d'Ivoire | MICS 2006 | 28.0 | 33.5 | 37.6 | 42.8 | 43.8 | 40.8 | 39.7 |
| Côte d'Ivoire | DHS 2011-12 | 31.3 | 35.1 | 36.8 | 40.3 | 45.4 | 44.6 | 46.9 |
| Côte d'Ivoire | MICS 2016 | 27.4 | 33.6 | 40.0 | 40.8 | 40.0 | 42.9 | 41.5 |
| Egypt | DHS 1995 | 98.1 | 98.3 | 97.0 | 95.8 | 96.7 | 97.2 | 96.8 |
| Egypt | DHS 2000 | 99.1 | 97.4 | 97.2 | 96.7 | 97.4 | 96.9 | 97.9 |
| Egypt | DHS 2003 | 96.8 | 97.4 | 97.3 | 96.5 | 96.4 | 96.5 | 98.0 |
| Egypt | DHS 2005 | 96.4 | 95.8 | 95.1 | 95.9 | 95.9 | 96.0 | 96.3 |
| Egypt | DHS 2008 | 93.8 | 93.6 | 94.3 | 95.7 | 97.1 | 96.8 | 96.7 |
| Egypt | DHS 2014 | 87.6 | 87.5 | 90 | 93.3 | 94.8 | 95.1 | 95.0 |
| Egypt | EHIS 2015 | 69.6 | 81.6 | 89.2 | 92.6 | 95.4 | 94.9 | 97.1 |
| Eritrea | DHS 1995 | 90.4 | 94.4 | 94.9 | 95.6 | 97 | 95.9 | 97.1 |
| Eritrea | DHS 2002 | 78.3 | 87.9 | 90.8 | 93.4 | 92.6 | 94.1 | 95.0 |
| Eritrea | EPHS 2010 | 68.8 | 79.1 | 84.9 | 88.4 | 90.4 | 93.0 | 93.1 |
| Ethiopia | DHS 2000 | 70.7 | 78.3 | 81.4 | 86.1 | 83.6 | 85.8 | 86.8 |
| Ethiopia | DHS 2005 | 62.1 | 73.0 | 77.6 | 78.0 | 81.2 | 81.6 | 80.8 |
| Ethiopia | DHS 2016 | 47.1 | 58.6 | 67.6 | 76.9 | 74.6 | 72.6 | 78.7 |
| Gambia | MICS 2005-06 | 79.9 | 78.2 | 77.2 | 78.4 | 79.5 | 77.7 | 74.2 |
| Gambia | MICS 2010 | 77.1 | 76.8 | 77.5 | 74.6 | 73.1 | 75.3 | 79.0 |
| Gambia | DHS 2013 | 76.3 | 74.1 | 73.5 | 73.9 | 76.8 | 74.9 | 75.9 |
| Gambia | MICS 2018 | 75.0 | 77.0 | 76.0 | 76.4 | 74.7 | 75.5 | 73.9 |
| Gambia | DHS 2019-20 | 72.6 | 71.7 | 74.8 | 71.3 | 72.1 | 71.6 | 74.0 |
| Ghana | DHS 2003 | 3.3 | 3.8 | 6.4 | 6.3 | 6.7 | 5.5 | 7.9 |
| Ghana | MICS 2006 | 1.4 | 2.3 | 2.7 | 5.7 | 5.7 | 5.1 | 7.4 |
| Ghana | MICS 2011 | 1.5 | 1.8 | 3.0 | 4.4 | 5.5 | 6.6 | 6.4 |
| Ghana | MICS 2017-18 | 0.6 | 1.5 | 1.8 | 3.2 | 3.0 | 3.6 | 4.9 |
| Guinea | DHS 1999 | 96.6 | 98.5 | 99.1 | 99.1 | 99.1 | 99.3 | 99.5 |
| Guinea | DHS 2005 | 89.3 | 94.6 | 96.6 | 97.4 | 98.6 | 98.1 | 99.5 |
| Guinea | DHS 2012 | 94.0 | 94.9 | 97.9 | 98.3 | 98.8 | 98.8 | 99.6 |
| Guinea | MICS 2016 | 94.5 | 96.5 | 97.3 | 97.3 | 96.6 | 99.1 | 98.7 |
| Guinea | DHS 2018 | 91.7 | 94.4 | 95.0 | 94.7 | 95.7 | 96.6 | 97.7 |
| Indonesia | 2013 RISKESDAS | 49 | 49 | 49 | 49 | 49 | 49 | 49 |
| Kurdistan | MICS 2011 * | 25.9 | 39.6 | 48.1 | 48.1 | 51.3 | 49.7 | 54.4 |
| Kurdistan | MICS 2018 * | 22 | 42.1 | 35.8 | 58.4 | 61 | 75.4 | 58.4 |
| Kenya | DHS 1998 | 26 | 32.2 | 40.4 | 40.9 | 49.3 | 47.4 | 47.5 |
| Kenya | DHS 2003 | 20.3 | 24.8 | 33.0 | 38.1 | 39.7 | 47.5 | 47.7 |
| Kenya | DHS 2008-09 | 14.6 | 21.1 | 25.3 | 30.0 | 35.1 | 39.8 | 48.8 |
| Kenya | DHS 2014 | 11.4 | 14.7 | 18.0 | 22.9 | 27.8 | 32.1 | 40.9 |
| Liberia | DHS 2013 | 31.1 | 39.8 | 50.1 | 60.3 | 60.3 | 63.3 | 72.4 |
| Liberia | DHS 2019-20 | 20.1 | 21.3 | 26.4 | 36.6 | 43.8 | 46.6 | 54.0 |
| Nigeria | DHS 2003 | 12.9 | 17.0 | 20.8 | 19.4 | 22.2 | 22.2 | 28.4 |
| Nigeria | MICS 2007 | 19.6 | 22.0 | 24.6 | 26.7 | 29.7 | 31.2 | 40.3 |
| Nigeria | DHS 2008 | 21.7 | 26.4 | 28.9 | 32.8 | 33.9 | 36.4 | 38.1 |
| Nigeria | MICS 2011 | 18.7 | 21.5 | 26.1 | 29.7 | 31.5 | 34.9 | 38.0 |
| Nigeria | DHS 2013 | 15.3 | 21.7 | 22.9 | 27.4 | 30.4 | 33.0 | 35.8 |
| Nigeria | MICS 2016-17 | 12.3 | 15.4 | 16.9 | 20.1 | 21.3 | 24.4 | 27.6 |
| Nigeria | DHS 2018 | 13.7 | 15.9 | 18.0 | 19.7 | 21.9 | 26.7 | 31.0 |
| Senegal | DHS 2005 | 24.8 | 28.0 | 28.4 | 30.1 | 30.5 | 30.3 | 30.6 |
| Senegal | DHS 2010-11 | 24 | 24.3 | 26.1 | 24.9 | 29.0 | 26.9 | 28.5 |
| Senegal | DHS 2014 | 21.1 | 21.5 | 27.9 | 28.2 | 24.9 | 27.2 | 25.7 |
| Senegal | DHS 2015 | 22.2 | 22.3 | 24.6 | 25.1 | 24.2 | 29.4 | 27.8 |
| Senegal | DHS 2016 | 20.6 | 22.6 | 22.5 | 23.3 | 25.0 | 22.2 | 26.0 |
| Senegal | DHS 2017 | 21.3 | 23.3 | 24.5 | 23.1 | 27.2 | 26.8 | 26.1 |
| Senegal | DHS 2018 | 21.4 | 21.9 | 25.1 | 25.4 | 22.9 | 23.8 | 24.5 |
| Senegal | DHS 2019 | 23.7 | 24.2 | 27.5 | 25.4 | 25.9 | 27.9 | 23.0 |
| Sierra Leone | MICS 2005 | 81.1 | 93.6 | 96.2 | 96.4 | 97.5 | 97.1 | 97.1 |
| Sierra Leone | DHS 2008 | 75.5 | 89.4 | 95.2 | 94.9 | 96.4 | 96.1 | 95.9 |
| Sierra Leone | MICS 2010 | 70.1 | 86.9 | 92.2 | 93.2 | 95.8 | 95.3 | 96.4 |
| Sierra Leone | DHS 2013 | 74.3 | 87.5 | 93.2 | 95.8 | 97.5 | 97.4 | 97.8 |
| Sierra Leone | MICS 2017 | 64.3 | 85.7 | 90.9 | 94.5 | 96.4 | 97.5 | 96.3 |
| Sierra Leone | DHS 2019 | 61.1 | 81.0 | 88.1 | 90.7 | 93.1 | 93.5 | 94.9 |
| Somalia | MICS 2006 | 96.7 | 97.9 | 97.9 | 98.8 | 98.9 | 97.9 | 99.1 |
| Somalia | MICS 2011 | 97.3 | 98.5 | 97.9 | 98.7 | 98.3 | 97.6 | 97.8 |
| Somalia | SHDS 2020 | 98.8 | 98.6 | 99.5 | 99.7 | 99.4 | 99.6 | 99.8 |
| Sudan | DHS 1989-90 | 86.8 | 89.7 | 88.6 | 89.7 | 89.0 | 89.0 | 90.9 |
| Sudan | MICS 2010 | 83.7 | 86.8 | 89.5 | 88.3 | 89.7 | 89.8 | 89.1 |
| Sudan | MICS 2014 | 81.7 | 85.7 | 87.6 | 88.0 | 86.6 | 91.4 | 91.8 |
| Tanzania | DHS 1996 | 13.5 | 15.9 | 19.6 | 20.8 | 18.7 | 21.3 | 22.2 |
| Tanzania | DHS 2004-05 | 9.1 | 13.7 | 15.2 | 16.0 | 16.0 | 18.8 | 22.9 |
| Tanzania | DHS 2010 | 7.1 | 11.0 | 11.7 | 19.1 | 21.6 | 22.2 | 21.5 |
| Tanzania | DHS 2015-16 | 4.7 | 7.3 | 8.1 | 12.8 | 13.4 | 15.4 | 18.7 |
| Togo | MICS 2006 | 1.3 | 2.5 | 6.2 | 6.5 | 9.4 | 10.0 | 9.5 |
| Togo | MICS 2010 | 1.1 | 2.4 | 3.7 | 4.7 | 6.4 | 5.2 | 6.7 |
| Togo | DHS 2013-14 | 1.8 | 2.2 | 3.8 | 4.9 | 6.5 | 8.6 | 10.2 |
| Togo | MICS 2017 | 1.4 | 1.9 | 2.4 | 3.8 | 4.1 | 4.7 | 5.4 |
| Uganda | DHS 2006 | 0.5 | 0.8 | 0.3 | 0.8 | 0.8 | 1.0 | 0.4 |
| Uganda | DHS 2011 | 1.0 | 0.8 | 1.9 | 2.1 | 1.3 | 1.7 | 1.9 |
| Uganda | DHS 2016 | 0.1 | 0.3 | 0.2 | 0.4 | 0.5 | 0.7 | 0.4 |
| Yemen | DHS 1997 | 19.3 | 22.2 | 21.3 | 22.9 | 23.6 | 25.1 | 25.0 |
| Yemen | DHS 2013 | 16.4 | 16.8 | 16.4 | 20.5 | 23.1 | 22.1 | 22.8 |
