# Supplementary material for: Modelling the impact of migration on FGM/C prevalence and risk in immigrant and diaspora populations
Source: PLoS One. 2026 Oct 13;21(10):e0360038. doi: 10.1371/journal.pone.0360038 (PMC13660914; doi:10.1371/journal.pone.0360038)
Supplement: Supplementary file 4 [file pone.0360038.s004.docx]

Supplemental Data Table 4: FGM/C prevalence from the latest nationally representative survey showing age cohort and national prevalence data, together with the prevalence by urban, education and wealth to calculate the Migration Selection Factor

| **Country** | **Survey** | **Age cohort prevalence %** | | | | | | | **National prevalence %** | | | | |
| --- | --- | --- | --- | --- | --- | --- | --- | --- | --- | --- | --- | --- | --- |
|  |  | **Age 15–19** | **Age 20–24** | **Age 25–30** | **Age 30 –34** | **Age 35–39** | **Age 40–44** | **Age 45–49** | **National Average** | **Urban** | **Secondary or Higher education** | **Top wealth quintile** | **Migration Selection Factor** |
| Cameroon | DHS 2004 | 0.4 | 2.5 | 1.6 | 1.1 | 1.2 | 1.8 | 2.4 | 1.40 | 0.90 | 0.40 | 0.70 | 0.48 |
| Côte d'Ivoire | MICS 2016 | 27.4 | 33.6 | 40.0 | 40.8 | 40.0 | 42.9 | 41.5 | 39.50 | 36.40 | 19.08 | 25.20 | 0.68 |
| Egypt | EHIS 2015 | 69.6 | 81.6 | 89.2 | 92.6 | 95.4 | 94.9 | 97.1 | 93.96 | 89.27 | 90.91 | 84.10 | 0.94 |
| Eritrea | EPHS 2010 | 68.8 | 79.1 | 84.9 | 88.4 | 90.4 | 93.0 | 93.1 | 88.73 | 86.43 | 82.23 | 83.83 | 0.95 |
| Ethiopia | DHS 2016 | 47.1 | 58.6 | 67.6 | 76.9 | 74.6 | 72.6 | 78.7 | 73.13 | 67.40 | 64.10 | 68.93 | 0.91 |
| Gambia | DHS 2019-20 | 72.6 | 71.7 | 74.8 | 71.3 | 72.1 | 71.6 | 74.0 | 75.56 | 74.04 | 73.42 | 67.18 | 0.95 |
| Ghana | MICS 2017-18 | 0.6 | 1.5 | 1.8 | 3.2 | 3.0 | 3.6 | 4.9 | 3.85 | 2.23 | 1.03 | 1.45 | 0.40 |
| Guinea | DHS 2018 | 91.7 | 94.4 | 95.0 | 94.7 | 95.7 | 96.6 | 97.7 | 96.40 | 95.78 | 93.45 | 96.03 | 0.99 |
| Indonesia | 2013 RISKESDAS | 49 | 49 | 49 | 49 | 49 | 49 | 49 | 49.00 | 56.00 | - | 53.00 | 1.11 |
| Kurdistan | MICS 2018 * | 22 | 42.1 | 35.8 | 58.4 | 61 | 75.4 | 58.4 | 29.48 | 18.68 | 19.83 | 17.65 | 0.63 |
| Kenya | DHS 2014 | 11.4 | 14.7 | 18.0 | 22.9 | 27.8 | 32.1 | 40.9 | 42.80 | 43.00 | 33.00 | 26.00 | 0.81 |
| Liberia | DHS 2019-20 | 20.1 | 21.3 | 26.4 | 36.6 | 43.8 | 46.6 | 54.0 | 44.00 | 35.35 | 31.40 | 24.70 | 0.56 |
| Nigeria | DHS 2018 | 13.7 | 15.9 | 18.0 | 19.7 | 21.9 | 26.7 | 31.0 | 23.47 | 30.61 | 29.11 | 30.43 | 1.28 |
| Senegal | DHS 2019 | 23.7 | 24.2 | 27.5 | 25.4 | 25.9 | 27.9 | 23.0 | 24.75 | 20.94 | 20.04 | 13.75 | 0.74 |
| Sierra Leone | DHS 2019 | 61.1 | 81.0 | 88.1 | 90.7 | 93.1 | 93.5 | 94.9 | 88.72 | 81.53 | 76.58 | 78.66 | 0.79 |
| Somalia | SHDS 2020 | 98.8 | 98.6 | 99.5 | 99.7 | 99.4 | 99.6 | 99.8 | 98.37 | 97.97 | 97.17 | 96.80 | 0.99 |
| Sudan | MICS 2014 | 81.7 | 85.7 | 87.6 | 88.0 | 86.6 | 91.4 | 91.8 | 81.10 | 85.50 | 93.75 | 82.10 | 0.98 |
| Tanzania | DHS 2015-16 | 4.7 | 7.3 | 8.1 | 12.8 | 13.4 | 15.4 | 18.7 | 14.28 | 7.58 | 4.15 | 7.33 | 0.44 |
| Togo | MICS 2017 | 1.4 | 1.9 | 2.4 | 3.8 | 4.1 | 4.7 | 5.4 | 4.38 | 3.23 | 1.35 | 2.28 | 0.52 |
| Uganda | DHS 2016 | 0.1 | 0.3 | 0.2 | 0.4 | 0.5 | 0.7 | 0.4 | 0.77 | 0.60 | 0.73 | 0.67 | 0.72 |
| Yemen | DHS 2013 | 16.4 | 16.8 | 16.4 | 20.5 | 23.1 | 22.1 | 22.8 | 20.55 | 21.45 | 22.80 | 20.25 | 1.02 |
