# Supplementary material for: Modelling the impact of migration on FGM/C prevalence and risk in immigrant and diaspora populations
Source: PLoS One. 2026 Oct 13;21(10):e0360038. doi: 10.1371/journal.pone.0360038 (PMC13660914; doi:10.1371/journal.pone.0360038)
Supplement: Supplementary file 5 [file pone.0360038.s005.docx]

Supplemental Data Table 5: Age of FGM/C Risk Boundaries

| **Population** | **q1 (25th)** | **50th** | **q3 (75th)** | **q3 + 1.5 x IQR** | **Source** |
| --- | --- | --- | --- | --- | --- |
| Cameroon | 6.00 | 7.00 | 10.00 | 16.00 | DHS2004 |
| Cote d’Ivoire | 3.00 | 5.00 | 8.00 | 15.50 | (UNFPA, 2020b) |
| Dawoodi Bohra | 6.00 | 6.50 | 7.00 | 8.50 | (Pinder *et al.*, 2024) |
| Egypt | 9.00 | 10.00 | 11.00 | 14.00 | (UNFPA, 2020b) |
| Eritrea | 0.50 | 1.50 | 4.00 | 9.25 | EPHS2010 |
| Ethiopia | 0.50 | 2.00 | 8.00 | 18.00** | (UNFPA, 2020b) |
| Gambia | 1.00 | 3.00 | 6.00 | 13.50 | (UNFPA, 2020b) |
| Ghana | 4.00 | 9.00 | 12.00 | 18.00** | (UNFPA, 2020b) |
| Guinea | 5.00 | 7.00 | 10.00 | 17.50 | (UNFPA, 2020b) |
| Indonesia | 0.50 | 1.00 | 2.00 | 4.25 | (Patel and Roy, 2016) |
| Kenya | 8.00 | 11.00 | 14.00 | 18.00** | (UNFPA, 2020b) |
| Kurdish | 4.00 | 5.00 | 7.00 | 11.50 | (UNFPA, 2020b) |
| Kuwait | 6.00 | 8.00 | 10.00 | 16.00 | (Chibber, El-saleh and Harmi, 2011) |
| Liberia | 4.00 | 9.00 | 14.00 | 18.00** | DHS2019-20 |
| Malay | 0.25 | 0.50 | 0.75 | 1.50 | (Morin, Callaghan and Issell, 2024) |
| Nigeria | 0.25 | 0.50 | 0.75 | 1.50 | (UNFPA, 2020b) |
| Saudi Arabia | 0.25 | 0.50 | 0.75 | 1.50 | (Rouzi *et al.*, 2019) |
| Senegal | 0.50 | 2.00 | 4.00 | 9.25 | (UNFPA, 2020b) |
| Sierra Leone | 10.00 | 12.00 | 15.00 | 18.00** | (UNFPA, 2020b) |
| Somalia | 5.00 | 7.00 | 9.00 | 15.00 | (SHDS, 2020) |
| Sudan | 5.00 | 7.00 | 8.00 | 12.50 | (UNFPA, 2020b) |
| Tanzania | 5.00 | 8.00 | 13.00 | 18.00** | (UNFPA, 2020b) |
| Togo | 5.00 | 8.00 | 12.00 | 18.00** | (UNFPA, 2020b) |
| UAE | 0.25 | 0.50 | 0.75 | 1.50 | (Al Awar *et al.*, 2020) |
| Uganda | 9.00 | 12.00 | 15.00 | 18.00** | (28 Too Many, 2013) |
| Yemen | 0.25 | 0.50 | 0.75 | 1.50 | (UNFPA, 2020b) |

** Records truncated at 18.
