# Supplementary material for: Modelling the impact of migration on FGM/C prevalence and risk in immigrant and diaspora populations
Source: PLoS One. 2026 Oct 13;21(10):e0360038. doi: 10.1371/journal.pone.0360038 (PMC13660914; doi:10.1371/journal.pone.0360038)
Supplement: Supplementary file 6 [file pone.0360038.s006.docx]

Supplemental Data Table 6: The results of the generalised gamma fitting showing the original observed quartiles, the method used to estimate each result, the fitted quartiles, the margin of error between the observed and fitted quartiles, the Stacy generalised gamma parameters d, p and a, and the cumulative proportion of the distribution at age 18.

| **Population** | **Original Quartiles** | | | **Fit method** | **Fitted quartiles** | | | **Margin of Error** | | | **Stacy GG parameters** | | | $\boldsymbol{F(18)}$ |
| --- | --- | --- | --- | --- | --- | --- | --- | --- | --- | --- | --- | --- | --- | --- |
|  | **25th** | **50th** | **75th** |  | **25th** | **50th** | **75th** | **25th** | **50th** | **75th** | $\boldsymbol{d}$ | $\boldsymbol{p}$ | $\boldsymbol{a}$ |  |
| Cameroon | 6 | 7 | 10 | approx. | 5.73 | 7.45 | 9.52 | -0.04 | 0.06 | -0.05 | 9.104 | 0.798 | 0.366 | 1.00 |
| Cote d'Ivoire | 3 | 5 | 8 | exact | 3.00 | 5.00 | 8.00 | - | - | - | 3.830 | 0.515 | 0.110 | 0.98 |
| Egypt | 9 | 10 | 11 | exact | 9.00 | 10.00 | 11.00 | - | - | - | 15.760 | 3.041 | 5.949 | 1.00 |
| Eritrea | 0.5 | 1.5 | 4 | exact | 0.50 | 1.50 | 4.00 | - | - | - | 1.360 | 0.333 | 0.028 | 0.97 |
| Ethiopia | 0.5 | 2 | 8 | near-exact | 0.50 | 2.00 | 7.92 | - | - | -0.01 | 15.443 | 0.015 | 1.11x10^-194^ | 0.86 |
| Gambia | 1 | 3 | 6 | exact | 1.00 | 3.00 | 6.00 | - | - | - | 0.637 | 2.722 | 10.228 | 1.00 |
| Ghana | 4 | 9 | 12 | approx. | 4.15 | 8.43 | 12.77 | 0.04 | -0.06 | 0.06 | 0.978 | 12.000 | 17.862 | 0.98 |
| Guinea | 5 | 7 | 10 | near-exact | 5.00 | 7.00 | 9.78 | - | - | -0.02 | 63.626 | 0.064 | 4.97x10^-47^ | 0.97 |
| Indonesia | 0.5 | 1 | 2 | near-exact | 0.50 | 1.00 | 1.99 | - | - | - | 30.886 | 0.031 | 7.45x10^-98^ | 1.00 |
| Kenya | 8 | 11 | 14 | exact | 8.00 | 11.00 | 14.00 | - | - | - | 2.552 | 3.427 | 13.895 | 0.95 |
| Kurdistan | 4 | 5 | 7 | approx. | 3.89 | 5.20 | 6.79 | -0.03 | 0.04 | -0.03 | 7.199 | 0.844 | 0.429 | 1.00 |
| Liberia | 4 | 9 | 14 | approx. | 4.03 | 8.88 | 14.17 | 0.01 | -0.01 | 0.01 | 0.877 | 8.236 | 20.793 | 0.90 |
| Nigeria | 0.25 | 0.5 | 0.75 | exact | 0.25 | 0.50 | 0.75 | - | - | - | 1.000 | 172.976 | 1.003 | 1.00 |
| Senegal | 0.5 | 2 | 4 | approx. | 0.51 | 1.92 | 4.16 | 0.02 | -0.04 | 0.04 | 0.524 | 7.888 | 7.687 | 1.00 |
| Sierra Leone | 10 | 12 | 15 | near-exact | 10.00 | 12.00 | 14.38 | - | - | -0.04 | 117.421 | 0.117 | 3.40x10^-25^ | 0.94 |
| Somalia | 5 | 7 | 9 | exact | 5.00 | 7.00 | 9.00 | - | - | - | 2.363 | 3.486 | 9.191 | 1.00 |
| Sudan | 5 | 7 | 8 | approx. | 5.07 | 6.82 | 8.20 | 0.01 | -0.03 | 0.02 | 2.339 | 12.000 | 9.501 | 1.00 |
| Tanzania | 5 | 8 | 13 | near-exact | 5.00 | 8.00 | 12.76 | - | - | -0.02 | 45.549 | 0.046 | 1.11x10^-65^ | 0.88 |
| Togo | 5 | 8 | 12 | exact | 5.00 | 8.00 | 12.00 | - | - | - | 2.571 | 1.046 | 3.879 | 0.93 |
| Uganda | 9 | 12 | 15 | exact | 9.00 | 12.00 | 15.00 | - | - | - | 2.952 | 3.339 | 14.116 | 0.91 |
| Yemen | 0.25 | 0.5 | 0.75 | exact | 0.25 | 0.50 | 0.75 | - | - | - | 1.000 | 172.976 | 1.003 | 1.00 |
