# Supplementary material for: Modelling the impact of migration on FGM/C prevalence and risk in immigrant and diaspora populations
Source: PLoS One. 2026 Oct 13;21(10):e0360038. doi: 10.1371/journal.pone.0360038 (PMC13660914; doi:10.1371/journal.pone.0360038)
Supplement: Supplementary file 8 [file pone.0360038.s009.docx]

**Modelling the impact of migration on FGM/C prevalence and risk in immigrant and diaspora populations [PONE-D-26-15067]**

**Response to Reviewer’s Comments**

**Note:**  line numbers throughout these responses refer to Manuscript (v4) (without tracked changes)

**Editor’s comments:**

**Comment:**

Please ensure that the above revisions are completed, upon which I shall be pleased to recommend that the paper be accepted and published.

**Response:**

Thank you so much for your endorsement. This is very encouraging. Please see all of the amendments below.

**Reviewer #2**

**Comment:**

The revised manuscript has improved substantially, and most of the concerns raised in the previous review have been addressed. However, two important issues remain unresolved and should be clarified before the manuscript can be published.

**Response:**

Thank you for your incredible comments and your support in sharpening this paper. You have been so incredibly helpful.

**Comment:**

The introduction of the Ethno-religious Loading Factor remains insufficiently justified. The revised manuscript applies an ELF of 2 to the Nigerian population and 0.5 to Egyptian girls growing up in the diaspora. However, these values appear to be selected on an ad hoc basis rather than derived from a transparent and reproducible estimation procedure.

For Nigeria, the doubling factor is inferred from a small number of local studies conducted in different destination countries and among populations that may not be representative of Nigerian migrants in the United States. For Egypt, differences in attitudes towards continuation of FGM/C are translated directly into a 50% reduction in risk, although attitudinal differences cannot automatically be interpreted as equivalent differences in behavioural prevalence or incidence.

More importantly, no general procedure is provided for estimating ELF across origin groups, and no sensitivity analysis is presented to show how the results change under alternative plausible values.

I therefore recommend one of these solutions

- Keeping ELF and 1) acknowledging the limitations 2) presenting estimates with and without ELF and assessing clearly the impact of inserting such a speculative coefficient
- either removing ELF from the main estimates and presenting it only as part of a clearly labelled sensitivity analysis.

The manuscript should also state more explicitly that the current ELF values are illustrative assumptions rather than empirically validated parameters.

**Response:**

I opted to implement your first option – to keep the ELF. I added additional text in several places in response to your comment.

In the literature review section I added text to explain the impact of ethnic/religious selection for both the Nigerian and the Egyptian cases.

In the Nigerian case, in lines 136 - 147, I added a more detailed description of the ethnic communities involved:

Since the prevalence in Nigeria varies significantly by ethnic origin and geographic location (state) [25], these results suggest that ethnic-specific selective migration was playing a significant role in these communities. A closer look at the ethnicity of the populations in these three studies confirms that suggestion. The population in Texas identified as Igbo, while the German study found that the majority of participants identified as Igbo or Yoruba. Prevalence among the Yoruba (54.5%) and Igbo (45.2%) are consistent with the higher figures observed in those two studies [25]. On the other hand, Farina and Ortensi [27] found that the majority of the Nigerian population in their Italian study originated from Edo State, which they note had a prevalence of over 50% at the time. While the findings of these three studies cannot be extrapolated to the entire Nigerian immigrant population, they are suggestive of an ethnic-specific selective migration pattern that should be considered.

Likewise, in lines 151 - 170, I added further explanation regarding the potential for attitude change post-migration and its impact on risk:

The significance of this selective migratory pattern on the prevalence of FGM/C in the Egyptian community in the US is profound, with Christian women almost three times more likely to believe that FGM/C should be discontinued than their Muslim counterparts – at a ratio of 76.1% to 29.5% respectively [30]. Within that context, Agboli *et al.* suggest that “[the] migration context appears to be a favourable platform for the change of views towards the practice of FGM” [31]. This view is shared by others who point to the opportunity that migration offers for reflection on one’s cultural practice [32–35]. Agboli *et al.* [31] proposed a five-stage model of attitude change that is triggered by migration. In their model, women shift from *Emotion Suppression,* in which they conform to community expectations prior to migration, through a process of *Awakening* that is precipitated by a series of turning points – moments of awareness – post migration, which trigger questioning of FGM/C. A *Clash* follows as women experience internal conflicts of loyalty between community norms and their well-being (especially when protecting their daughters). In time, women actively reconstruct their identity by reconciling traumatic memories and rejecting harmful norms, leading to a *Re-appropriation of Self* and eventually *Reconciliation* as they forgive their mothers and maintain cultural ties, while rejecting the practice of FGM/C, mirroring Berry’s [36] integration acculturation strategy. While the process that Agboli *et al.* [31] describes could be applied to any immigrant population, the attitudinal data and the selective migratory trend within the Egyptian population suggests a special case.

Then in the methodology section I pointed to the speculative nature of the factor in lines 306 - 324:

These socio-economic influences, however, pale in comparison to the ethnic-specific nature of the practice [58,59]. To adjust for the impact of ethnic- and religious-based selective migration, an additional factor was introduced to the extrapolation equation. The *Ethno-religious Loading Factor* (ELF) was used to assess the potential impact of ethnic and religious selection in two populations. A sensitivity analysis was conducted, initially assuming no impact resulting from ethnic and religious selection (ELF = 1). This was followed by a scenario where ethnic and religious selection was considered to have an impact. In that scenario, the factor was set to double (ELF = 2) the age-disaggregated prevalence in the Nigerian population to account for the doubling effect of selective migration evident from previous studies. Similarly, to account for religiously driven selective migration in the Egyptian population, the factor was set to halve the risk to Egyptian girls growing up in the diaspora (ELF = 0.5). In the Nigerian case, ethnic and geographic selective migration likely impacted both the women and girls migrating with FGM/C and the risk to Nigerian girls growing up in the US. On the other hand, the prevalence of FGM/C in Egypt does not differentiate by religion [30]; however, in the migratory context, unsupportive attitudes likely result in a drop in risk for Egyptian girls growing up in the US. These ELF settings are speculative and are included to demonstrate the potential impact of ethnic and religious selective migration. The final results are presented in two forms, first with the ELF and then without the ELF to clearly demonstrate the impact.

Then in the results section, I included a third table with a set of results without the ELF, thus creating the sensitivity analysis suggested, in lines 558 - 567:

**The impact of the ELF**

To assess the impact of the *Ethno-religious Loading Factor* (ELF), Table 3 presents the same results as Table 2, but removes its effects (ELF=1) from all populations. Removing the effects of the ELF from the results reduces the living-with population by between 9.7% and 12.1% depending on the PIF setting, while it increases the at-risk population by 17.8% across all PIF scenarios.

**Table 3: Results based on the generalised gamma distribution results shown in S6 Table, with the ELF = 1 for all populations and the Prevention Impact Factor was set to 0, 0.25, 0.50, and 0.75.**

| **Population Segment** | **Labelling**  **PIF = 0** | **Deterrence**  **PIF = 0.25** | **Desistance**  **PIF = 0.50** | **Community abandon-ment**  **PIF = 0.75** |
| --- | --- | --- | --- | --- |
| 1a: Foreign-born and cut before migration | 348,231 | 348,231 | 348,231 | 348,231 |
| 1b: Foreign-born and cut after migration | 36,995 | 27,746 | 18,498 | 9,249 |
| 1c: Foreign-born & potentially at risk after migration | 8,122 | 6,092 | 4,061 | 2,031 |
| 2a: Native-born and cut | 107,025 | 80,269 | 53,513 | 26,756 |
| 2b: Native-born and potentially at risk | 41,883 | 31,412 | 20,942 | 10,471 |
| **Population impacted by FGM/C (1a + 1b + 1c + 2a + 2b)** | **542,256** | **493,750** | **445,244** | **396,737** |
| Living with FGM/C (1a + 1b + 2a) | 492,251 | 456,246 | 420,241 | 384,236 |
| At risk of FGM/C (1c + 2b) | 50,005 | 37,504 | 25,003 | 12,501 |
| Estimate of FGM/C that occurred while US-based (1b + 2a) | 144,020 | 108,015 | 72,010 | 36,005 |
| Study population (SP) | 1,248,776 | 1,248,777 | 1,248,778 | 1,248,779 |
| **Population not impacted by FGM/C (SP – Impacted)** | **706,520** | **755,027** | **803,535** | **852,042** |

Finally, I added a sentence to the discussion in this regard in lines 571 - 577:

The modelling developed in this paper attempted to quantify that impact and, in so doing, demonstrated that Jones et al. [13] and Goldberg *et al.* [14] likely overestimated the impacted population by 12.9% (n = 79,817) and 6.3% (n = 36,388), respectively, when setting the PIF to represent the hypothesised deterrence scenario (PIF = 0.25) and considering the hypothesised impact of ethno-religious selective migration. Removing the ELF from the estimation suggests that Jones et al. [13] and Goldberg *et al.* [14] likely overestimated the impacted population by 20.4% (n = 126,845) and 14.5% (n = 83,416), respectively.

**Comment**:

The manuscript still does not distinguish consistently between women and girls living with FGM/C, girls at risk of FGM/C, and the broader population described as “impacted.” This is particularly evident in Table 1, where the sum of those estimated to be living with FGM/C and those estimated to be at risk is reported as “estimated FGM/C prevalence.”

This terminology is not conceptually correct. Girls who are currently at risk have not necessarily undergone FGM/C and may never do so. Therefore, the combined category of “living with FGM/C” plus “at risk” cannot be interpreted as prevalence.

It would be more appropriate to report separately:

- -estimated prevalence of FGM/C;
- -estimated number or proportion currently at risk;
- -total population potentially impacted, if the authors wish to retain this broader category

**Response:**

I have made several edits throughout the document to clarify the terms impacted, living-with and at-risk. Most notably, in the introduction, in lines 40 - 43, I replaced impacted with living with in describing Yoder’s extrapolation:

The extrapolation method was originally proposed by Yoder *et al*. [7] for estimating the number of women living with FGM/C in Africa. At the time, Yoder *et al*. [7] argued that the extrapolation method they used was ill-suited to the estimation of the population living with FGM/C in immigrant contexts …

I then also removed the prevalence rate in the impacted population from the results tables in lines 557 and 567.

**Comment:**

Relatedly, expressions such as “FGM/C that occurred while US-based” are too definitive, because the model does not observe whether FGM/C actually occurred after migration.

It probabilistically allocates expected cases across pre-migration and post-migration periods. The results should therefore be described consistently as model-based estimates or expected numbers rather than observed events.

These distinctions are essential because the current terminology risks overstating the precision and empirical status of the findings.

**Response:**

I have softened the language regarding FGM/C occurrence in the US in the results tables to read “Estimate of FGM/C that occurred while US-based (1b + 2a)” in lines 557 and 567.
