# Supplementary material for: Modelling the impact of migration on FGM/C prevalence and risk in immigrant and diaspora populations
Source: PLoS One. 2026 Oct 13;21(10):e0360038. doi: 10.1371/journal.pone.0360038 (PMC13660914; doi:10.1371/journal.pone.0360038)
Supplement: Supplementary file 9 [file pone.0360038.s008.docx]

**Modelling the impact of migration on FGM/C prevalence and risk in immigrant and diaspora populations [PONE-D-26-15067]**

**Response to Reviewer’s Comments**

**Note:** line numbers throughout these responses refer to Manuscript (v3) (without tracked changes)

**Editor’s comments:**

**Comment:**

If applicable, we recommend that you deposit your laboratory protocols in protocols.io to enhance the reproducibility of your results. Protocols.io assigns your protocol its own identifier (DOI) so that it can be cited independently in the future …

**Response:**

The R code has been uploaded to protocols.io , and the DOI is now included in the referencing of the paper. For ease of review, the DOI is <https://www.protocols.io/blind/A4009179707A11F1AE1B0A58A9FEAC02>

**Journal requirement:**

**Comment:**

Please ensure that your manuscript meets PLOS ONE's style requirements, including those for file naming.

**Response:**

The following style edits were completed:

- All level 1 headings have been changed to bold type 18pt font.
- All level 2 headings have been changed to bold type 16pt font.
- All level 3 headings have been changed to bold type 14pt font.
- All figure citations have been changed to Fig 1, Fig 2.
- All figure captions have been reformatted.
- All table captions have been reformatted.
- Table numbering has been updated.

**Comment:**

Please include captions for your Supporting Information files at the end of your manuscript, and update any in-text citations to match accordingly.

**Response:**

Captions for the Supporting Information files have been added to the manuscript after references.

**Additional Editor Comments:**
Please make sure that reviewer 2's insightful comments are all adequately addressed, given the importance of the topic and that of the analyses in this case.

**Response:**

I have fully addressed the comments made by Reviewer #2, as shown below.

**Reviewer #1**

**Comment:**
One question did occur to me in terms of migration timing and the impact of selective migration. Looking at involuntary mobility, might the formula need adjusting when assessing women and girls held in refugee camps for many years? There are refugee camps where migrant groups may be very heterogeneous or may be influenced by outside factors over time.

**Response:**

The point regarding women held in refugee camps is important but cannot be modelled since the population data does not include that information. I have, however, referred to this in the limitations as follows on lines 573 – 576:

Three primary limitations are identified. The generalised gamma distribution modelling assumes unimodal age distributions of FGM/C. The impact of long-term residence in refugee camps prior to migration has not been accounted for in the modelling. Furthermore, this paper has not modelled the impact of circular migration or acculturation …

**Comment:**

Another question is whether women and girls seeking asylum fit into the refugee involuntary migrant group.

**Response:**
This comment is more easily dealt with in the main text. I have added a reference in this regard as follows in lines 113 - 116:

Since refugee resettlement draws its participants from the latter group, it can be argued that Ortensi *et al.’s* assertion holds in relative terms. Likewise, a similar pattern can be observed among asylum seekers [21].

**Reviewer #2**

**Comment:**

The literature review requires clarification, as it currently tends to conflate studies with different objects of estimation and different methodological approaches. The manuscript states that the “Extrapolation-of-FGM/C-Countries’ Prevalence-Data method” was developed by Yoder et al. and Van Baelen et al. and later codified by EIGE. This formulation is misleading. Yoder et al. and Van Baelen et al. primarily estimate FGM/C prevalence among women and girls, whereas EIGE’s step-by-step guide focuses specifically on estimating girls at risk of FGM/C. Relatedly, the manuscript attributes to Yoder et al. and Van Baelen et al. a modeling structure that includes a migration and acculturation parameter, m. This does not appear to be accurate. In particular, Van Baelen et al. do not use such a coefficient. Van Baelen said that “the estimations provided for this study follow the standard for baseline estimation proposed by EIGE” (that does not include m) and never mention acculturation, neither does Yoder.
This is not a minor terminological issue: it affects the way the manuscript reconstructs the existing literature and the extent to which the proposed model can be presented as an extension of previous work. The author should revise the literature review to distinguish more carefully between: prevalence estimation among women and girls; estimation of girls currently or prospectively at risk. At present, the review risks misrepresenting the methodological lineage of the field. The use of m and the median age for cutting is typical of the EIGE approach that explicitly refers to risk.

**Response:**

These comments have been fully addressed with a complete rewrite of that section in lines 38 to 91.

The method most often used for estimating the scale and distribution of the FGM/C-impacted population in diaspora contexts is the Extrapolation-of-FGM/C-Countries’-Prevalence-Data method [6]. The extrapolation method was originally proposed by Yoder *et al*. [7] for estimating the number of women impacted by FGM/C in Africa. At the time, Yoder *et al*. [7] argued that the extrapolation method they used was ill-suited to the estimation of the impacted population in immigrant contexts, pointing to selective migration, intra-country ethnic diversity, and declining prevalence in countries of origin as issues that the model did not address and which would likely impact the accuracy of the extrapolated results. Notwithstanding these concerns, Van Baelen *et al.* [8] applied the method in Europe. In this form, the extrapolation method consisted of two variables: FGM/C prevalence (Pr) in the country of origin and the first-generation immigrant population (p) in the country under examination. The product of these variables produced an estimate of the number of immigrants already living with FGM/C. The European Institute for Gender Equality (EIGE) adapted the extrapolation method [9] to estimate the number of first and second-generation girls who were at risk of FGM/C in Europe. In so doing, the EIGE added a third variable to the equation: an estimation of the impact of migration and acculturation (m). In the EIGE method, two scenarios are calculated: in the high-risk scenario, migration and acculturation are assumed to have no impact on risk (m = 0), while in the low-risk scenario, native-born girls are considered to be at half the risk (m = 0.5) of their foreign-born counterparts.

Kawous *et al.* [10], Nestlinger *et al.* [11] and Stegner et al. [12] effectively combined these two approaches: to calculate the number of women and girls living with FGM/C, they used van Baelen et al.’s [8] approach in which migration and acculturation are not considered, while they turned to the EIGE method [9] to estimate the number of girls at risk. Stegner et al. [12] followed the EIGE assumption that the risk to native-born girls was half that of foreign-born girls (m = 0.5). On the other hand, Kawous *et al.* [10] conducted a sensitivity analysis setting m at various levels between 0 and 1, whereas Nestlinger *et al.* [11] calculated country-specific values for m based on survey data. While there were additional nuances in each of these studies, their combined approach to calculating the potentially impacted population (both those living with FGM and those at risk) could broadly be expressed in the following extrapolation formula:

$$IP=\sum_{n=1}^{c} \left( \Pr_{\left( x,y \right),n} \times p_{\left( x,y \right),n} \times(1-m_{n} \right))$$

Where:

IP = the population impacted by FGM/C (both living with and at risk)

$\Pr_{\left( x,y \right),n}$ = the FGM/C prevalence disaggregated by five-year age cohorts (x,y) in a specific country-of-origin (n)

$p_{\left( x,y \right),n}$ = the first- and second-generation study population in the country-of-residence associated with a specific country-of-origin (n) disaggregated by five-year age cohorts (x,y)

(x,y) = five-year age cohorts 0-4, 5-9, 10-14, 15-19, 20-24 etc.

$m_{n}$ = a composite variable indicating the migration and acculturation impact in a range from 0 (no impact) to 1 (total impact)

c = the number of countries of origin under investigation

Previous studies in the United States (US), on the other hand, were based on an even simpler formula: Jones *et al.* [13] simply multiplied the study population by the national average prevalence in countries of origin to establish the total number of women and girls living with or at risk of FGM/C. Goldberg et al. [14] split the study population at age 20 and applied the 15-19 prevalence to those younger than 20 and the national average to the rest. Neither made any attempt to consider the impact of migration and acculturation.

In the formulation above, the single variable (m) models the impact of both migration and acculturation. This paper will explore the profound heterogeneity hidden within that single variable (m). However, in so doing, the paper will seek to model the impact of migration alone. While migration clearly also influences acculturation strategies and experiences, modelling the impact of acculturation is beyond the scope of this study.

**Comment:**

The use of a generalized gamma distribution to model the timing of FGM/C is one of the most interesting methodological innovations in the manuscript. It is preferable to fixed age-threshold approaches, because age at FGM/C varies substantially across countries and communities. However, the fitting procedure is not described in sufficient detail for replication. The manuscript states that the parameters were estimated through a three-stage process involving analytical root-finding, dense numerical search, and bounded nonlinear optimization. This is not enough for readers to evaluate the robustness of the estimates.

The author should provide:

- the exact objective function used;
- parameter constraints;
- starting values or search ranges;
- software or packages used;
- goodness-of-fit criteria;
- treatment of countries with quartiles equal or close to zero;
- code or pseudocode in the supplementary material.

Without these details, the generalized gamma estimates remain difficult to assess.
The gamma-based approach assumes a unimodal distribution of age at FGM/C. The author acknowledges that some countries may have wider or potentially multimodal distributions, including Liberia. However, this issue deserves more attention because the paper’s main empirical conclusion depends heavily on the shape of the age-at-cutting distribution.

The author should conduct sensitivity analyses and provide them in the appendix.

**Response:**

These comments have been addressed in several ways. First, the comment caused the author to reevaluate and update the R code used to fit the generalised gamma distributions to the age of FGM/C data. The revised code was then uploaded to protocols.oi. The protocols.oi entry then specifically addresses each of the bullet-pointed items above.

Second, the resultant Probability Density Function graphs and data outputs have been added to the supplemental material. These results are reported in the narrative in lines 470 to 475 as follows:

The results, as shown in S6 Table, show that 95.2% of the fitted quartiles fell within the 95% confidence level of the original quartiles, and all fell within the 90% confidence level. Turning to the case of Liberia, the fitted quartiles were all within the 99% confidence level of the original quartiles, suggesting that the gamma distribution was able to match the observed distribution despite the potential bi-modal nature of the practice.

Finally, the reviewers’ comments were addressed by reworking the text in lines 348 to 361:

The Stacy parameters $\left( d, p, a \right)$ were estimated from the quartiles in S6 Table using a three-stage process in R version 4.2.2 [58]. First, an analytical root-finding approach solved for a gamma shape that reproduced the observed interquartile ratios exactly using the uniroot function in R (n=10). If that approach failed, an attempt was made to identify a gamma curve with a near-exact fit (a maximum relative error of < 10% across all quartiles) using a dense numeric search of 800 parameter combinations that minimised the quartile ratio error (n=5). The remaining cases (n=6) were fitted to a gamma curve by bounded nonlinear optimisation using the R function nlminb. To prevent the optimiser from getting stuck in local minima, it was initialised with five diverse starting parameters. The objective function minimised the weighted sum of squared log-relative errors between the fitted quartiles and the observed quartiles, with a 50% higher weight applied to the median to prioritise central accuracy. A lower bound of 10^–12^ was used within the log-space transformations to prevent log(0) errors in cases where the input data approached zero (Nigeria, Yemen, Eritrea, Ethiopia, Indonesia, and Senegal).

**Comment**:

The expanded model includes several factors, including the Migration Selection Factor, Ethno-religious Loading Factor, Prevention Impact Factor, Circular Migration Risk Factor, and Acculturation Impact Factor. Conceptually, these dimensions may be relevant. Empirically, however, several are not implemented. For example, the Ethno-religious Loading Factor is set to 1, although the manuscript argues that ethno-religious selection could be very important. The Circular Migration Risk Factor is conceptualized but not quantified. The Acculturation Impact Factor is set to zero because the paper does not offer a method to estimate it. As several proposed innovations are not estimated, and in some cases it is not even clear what data would be required or how the parameters could be operationalized this raises a fundamental question: if the paper does not offer a method for estimating a parameter, what is the analytical value of including it in the model? The sentence “Since this paper does not offer a method by which to estimate the acculturation effect, the AIF was set accordingly (AIF = 0)” is particularly problematic. If the author cannot explain how the acculturation effect should be estimated, why should it be part of the formal model? At present, the model risks appearing more empirically complete than it actually is.

If the author wishes to retain these additional parameters, the manuscript should clearly distinguish between:

- components actually estimated in the present analysis;
- components included only as theoretical extensions;
- components explored through sensitivity analysis.

And provide a clear assessment of:

- what data would be required;
- whether such data currently exist;
- how the parameters could be estimated;
- why they are not implemented in the present study;
- how sensitive the results would be to plausible values;
- worked examples, even using hypothetical or simulated data.

Otherwise, these components should be removed from the main model and discussed only briefly as possible directions for future research. In their current form, they risk remaining pure abstraction without a clear horizon of empirical feasibility.

**Response:**

This comment was responded to in three ways. First, the Ethno-religious Loading Factor has now been included in the calculation, and its impact is reported in lines 450 to 454:

Building on these baseline estimates, to account for socio-economic selective migration by including Ortensi *et al*.’s [19] *selection hypothesis coefficient*, further reduced the high road estimate to 542,225, and the low road estimate to 467,801. Accounting for ethno-religious selective migration, through the inclusion of an *Ethno-religious Loading Factor* (ELF), increased the high road estimate to 548,243, and the low road estimate to 477,816.

Second, the Circular Migration Risk Factor and Acculturation Impact Factor were removed from the new extrapolation formula in lines 403 to 424:

To account for the impact of migration on the prevalence and risk of FGM/C in immigrant and diaspora populations, the extrapolation formula was updated as follows:

Those living with FGM/C were estimated using the following calculation:

$$\mathrm{LW}\mathbf{=}\sum_{n=1}^{c} \left( \Pr_{\left( x,y \right),n} \times p_{\left( x,y \right),n} \times s_{n}\times\mathrm{ELF}_{nav,n}\times PM \right)$$

$$+\sum_{n=1}^{c} \left( \Pr_{\left( x,y \right),n} \times p_{\left( x,y \right),n} \times s_{n}\times\mathrm{ELF}_{nav,n}\times SM\times\left( 1-PIF \right) \right)$$

While those at risk of FGM/C were estimated using the following calculation:

$$\mathrm{AR}\mathbf{=}\sum_{n=1}^{c} \left( \Pr_{\left( x,y \right),n} \times p_{\left( x,y \right),n} \times s_{n}\times\mathrm{ELF}_{nav,n}\times FR\times\left( 1-PIF \right) \right)$$

**The sum of those two results produced the impacted population.**

Finally, the Circular Migration Risk Factor and Acculturation Impact Factor were included in a new future research section in lines 581 to 632.

**Accounting for circular migration**

While it is clear that circular migration poses a risk to both native- and foreign-born girls during visits to their parents’ countries of origin [40,64–69], the extent of these risks is difficult to quantify. Notwithstanding the challenge of quantifying the scale, it is possible to establish the modelling framework. From a modelling perspective, a *Circular Migration Risk Factor* (CMRF) could be introduced. The CMRF would be an estimate of the proportion of girls who were exposed to the risks associated with circular migration at the ethnic community level. Setting the *Circular Migration Risk Factor* to 0 effectively removes its influence from the equation, while setting it to 1 removes the protective impact of the *Prevention Impact Factor (PIF)*. A setting between those two extremes removes the protective impact from the subset of girls who are exposed to risk in countries of origin as a result of circular migration.

**Accounting for acculturation**

The specifics of migration also influence individuals’ acculturation strategies and experiences. On the one hand, migration potentially provides a catalyst for attitude change through exposure to new knowledge, affecting acculturation responses [32,65,70–72]. On the other hand, migration timing determines the extent of exposure an individual has to the destination context [32,33,35–37,73]. Moreover, migration networks and destination locations affect the extent to which individuals are located in closed communities, which, combined with migration aspirations, suggest different acculturation strategies and experiences [15,40,74–76]. The effects of acculturation, once they are estimated, could be modelled using the *Acculturation Impact Factor* (AIF).

**An expanded extrapolation formula**

Bringing these additions together, the expanded extrapolation formula could be presented as follows:

Those living with FGM/C could be estimated using the following calculation:

$$\mathrm{LW}\mathbf{=}\sum_{n=1}^{c} \left( \Pr_{\left( x,y \right),n} \times p_{\left( x,y \right),n} \times s_{n}\times\mathrm{ELF}_{nav,n}\times PM \right)$$

$$+\sum_{n=1}^{c} \left( \Pr_{\left( x,y \right),n} \times p_{\left( x,y \right),n} \times s_{n}\times\mathrm{ELF}_{nav,n}\times SM\times(\left( \left( 1-\mathrm{CMRF}_{n} \right)\times\left( 1-PIF \right) \right)+ \mathrm{CMRF}_{n})\times(1-AI) \right)$$

While those at risk of FGM/C could be estimated using the following calculation:

$$\mathrm{AR}\mathbf{=}\sum_{n=1}^{c} \left( \Pr_{\left( x,y \right),n} \times p_{\left( x,y \right),n} \times s_{n}\times\mathrm{ELF}_{nav,n}\times FR \times(\left( \left( 1-\mathrm{CMRF}_{n} \right)\times\left( 1-PIF \right) \right)+\mathrm{CMRF}_{n})\times(1-AI) \right)$$

**The sum of those two results would produce the impacted population.**

**Comment:**

The Prevention Impact Factor is linked to criminological concepts such as labelling, deterrence, desistance, and community abandonment. This is an interesting theoretical framing. However, the numerical values assigned to these scenarios—0, 0.25, 0.50, and 0.75—appear arbitrary. The author should present these values explicitly as hypothetical scenarios rather than empirically grounded estimates. The sensitivity analysis is useful, but the interpretation should be cautious, especially because the PIF substantially affects the estimated number of girls still at risk.

**Response:**

The text was edited to explicitly state that the values were hypothetical scenarios in lines 395 to 401:

In this modelling, the *Prevention Impact Factor* settings were aligned with the criminology theory to create hypothetical scenarios as follows: Labelling (PIF = 0); deterrence (PIF = 0.25); desistance (PIF = 0.5). These three theories operate within an independent decision-making context; however, since decision-making regarding FGM/C operates as an interdependent process, it would hypothetically take community-led collective abandonment to achieve a higher *Prevention Impact Factor* (PIF = 0.75).

**Comment**:

The manuscript should revise its terminology around selective migration. Ortensi et al. do not refer to a “Migration Selection Factor” in the way the manuscript suggests. Rather, they discuss a correction according to the selection hypothesis. The manuscript should either use Ortensi et al.’s terminology or explicitly state that “Migration Selection Factor” is the author’s own label for this correction.

**Response**:

The terminology was aligned with Ortensi et al throughout the paper

**Comment:**

The manuscript argues that previous estimates overstate the number of girls at risk and understate the number of women and girls already living with FGM/C. This is a potentially important finding. However, because this is driven mainly by assumptions and scenarios that cannot be verified the policy conclusion should be presented with greater attention to uncertainty.

**Response**:

The language throughout the discussion has been adapted to take this into account. For example, in lines 529 to 543:

Previous estimates of FGM/C in the US and Europe likely overestimated the impacted population because they did not accurately account for the profound impact of migration. The modelling developed in this paper attempted to quantify that impact and, in so doing, demonstrated that Jones et al. [13] and Goldberg *et al.* [14] likely overestimated the impacted population by 12.9% (n = 79,817) and 6.3% (n = 36,388), respectively, when setting the PIF to represent the hypothesised deterrence scenario (PIF = 0.25). Furthermore, Goldberg *et al.* reported their results as the number of women and girls who “were at risk of FGM/C or its consequences” [14], which led many to interpret their data as “500,000 women and girls in the United States are at risk of genital mutilation” [62]. The modelling in this paper suggests that this characterisation of the data could not be further from the truth. Segmenting the impacted population into those who are living with FGM/C and those who remain at risk showed that an overwhelming 94.1% (n = 508,953) were likely already living with FGM/C, while only 5.9% (n = 31,826) were thought to be at risk. The primary driver of that shift from at-risk to living-with was modelling the distribution of ages at which FGM/C occurs in each ethnic community.

**Comment:**

The distinction between demographic and ethnic selection should also be reconsidered. The statement that “selective migration is not only a factor of demographics, but it also shapes the ethnic composition of the immigrant population” is not entirely convincing, since ethnic composition, when mediated by migration patterns, is itself a demographic characteristic of the migrant population.

**Response:**

“Demographic selection” was renamed as “socio-economic selection” throughout the manuscript to better represent the mechanism at play and distinguish it from “ethno-religious selection”. For example, in lines 106 to 109:

This observation supports Ortensi *et al*.’s [18,19] *selection hypothesis,* which accounts for the impact of socio-economic-driven selective migration by moderating prevalence to account for a more educated, wealthier, and urban immigrant population.

**Comment:**

The Nigerian example: The manuscript interprets higher FGM/C prevalence among Nigerian migrants mainly as evidence of ethnic selective migration. However, at least in the Italian case, this pattern is also strongly related to geographical selection, as many Nigerian migrants come from Edo State. The manuscript should therefore avoid framing the Nigerian example only in ethnic terms and should acknowledge that regional/geographical selection may be equally or more relevant in some destination countries.

**Response**:

The Nigerian selection was reframed as both ethnic and geographic. For example, in the lines 136 to 138:

Since the prevalence in Nigeria varies significantly by ethnic origin and geographic location (state) [25], these results suggest that selective migration was playing a significant role in these communities.

**Comment:**

The manuscript should clarify why the generalized gamma distribution was preferred over simpler alternatives, such as empirical cumulative distributions based on DHS/MICS age categories.

**Response**:

I have added a couple of sentences clarifying why I used the generalised gamma rather than a simpler alternative in lines 343 to 347:

That flexibility enabled a standard set of parameters $(d, p, a)$ to describe the proportional distribution of risk of FGM/C across the age of FGM/C window for all populations of interest. Rather than using a simpler alternative, such as an empirical cumulative distribution based on MICS and DHS age categories, fitting the generalised gamma distribution provides a continuous, statistically smoothed probability curve that enables precise estimation at the exact age milestones associated with migration history.

**Comment:**

The terminology “age-desegregated” appears to be used where “age-disaggregated” may be intended.

**Response**:

Age-desegregated has been corrected to age-disaggregated throughout the manuscript.

**Comment:**

The author should review the use of terms such as “prevalence,” “risk,” “impacted,” “living with,” and “at risk” to ensure that they are used consistently throughout the paper.

**Response**:

These have been reviewed throughout the manuscript.

**Additions to the supplemental data:**

Two tables were added to the supplemental data file.

S5 Table. Age of FGM/C Risk Boundaries.

S6 Table. The results of the generalised gamma fitting showing the original observed quartiles, the method used to estimate each result, the fitted quartiles, the margin of error between the observed and fitted quartiles, the Stacy generalised gamma parameters d, p and a, and the cumulative proportion of the distribution at age 18.
