# Supplementary material for: Modelling the impact of migration on FGM/C prevalence and risk in immigrant and diaspora populations
Source: PLoS One. 2026 Oct 13;21(10):e0360038. doi: 10.1371/journal.pone.0360038 (PMC13660914; doi:10.1371/journal.pone.0360038)
Supplement: Peer review history

# Peer review: Decision Letter 0

**DOI:** 10.1371/journal.pone.0360038.r001

**Authors:** Surajit Sen Surajit Sen

22 May 2026

Dear Dr. Callaghan,

Thank you for submitting your manuscript to PLOS ONE. After careful consideration, we feel that it has merit but does not fully meet PLOS ONE’s publication criteria as it currently stands. Therefore, we invite you to submit a revised version of the manuscript that addresses the points raised during the review process.

Please submit your revised manuscript by Jul 06 2026 11:59PM. If you will need more time than this to complete your revisions, please reply to this message or contact the journal office at plosone@plos.org. When you're ready to submit your revision, log on to https://www.editorialmanager.com/pone/ and select the 'Submissions Needing Revision' folder to locate your manuscript file.

- A letter that responds to each point raised by the academic editor and reviewer(s). You should upload this letter as a separate file labeled 'Response to Reviewers'.
- A marked-up copy of your manuscript that highlights changes made to the original version. You should upload this as a separate file labeled 'Revised Manuscript with Track Changes'.
- An unmarked version of your revised paper without tracked changes. You should upload this as a separate file labeled 'Manuscript'.

If you would like to make changes to your financial disclosure, please include your updated statement in your cover letter. Guidelines for resubmitting your figure files are available below the reviewer comments at the end of this letter.

If applicable, we recommend that you deposit your laboratory protocols in protocols.io to enhance the reproducibility of your results. Protocols.io assigns your protocol its own identifier (DOI) so that it can be cited independently in the future. For instructions see: https://journals.plos.org/plosone/s/submission-guidelines#loc-laboratory-protocols. Additionally, PLOS ONE offers an option for publishing peer-reviewed Lab Protocol articles, which describe protocols hosted on protocols.io. Read more information on sharing protocols at https://plos.org/protocols?utm_medium=editorial-email&utm_source=authorletters&utm_campaign=protocols.

As the corresponding author, your ORCID iD is verified in the submission system and will appear in the published article. PLOS supports the use of ORCID, and we encourage all coauthors to register for an ORCID iD and use it as well. Please encourage your coauthors to verify their ORCID iD within the submission system before final acceptance, as unverified ORCID iDs will not appear in the published article. *Only*  the individual author can complete the verification step; PLOS staff *cannot*  verify ORCID iDs on behalf of authors.

We look forward to receiving your revised manuscript.

Kind regards,

Surajit Sen, PhD

Academic Editor

PLOS One

Journal requirements:

When submitting your revision, we need you to address these additional requirements.

1. Please ensure that your manuscript meets PLOS ONE's style requirements, including those for file naming. The PLOS ONE style templates can be found at

https://journals.plos.org/plosone/s/file?id=wjVg/PLOSOne_formatting_sample_main_body.pdf and

https://journals.plos.org/plosone/s/file?id=ba62/PLOSOne_formatting_sample_title_authors_affiliations.pdf

2. Please include captions for your Supporting Information files at the end of your manuscript, and update any in-text citations to match accordingly. Please see our Supporting Information guidelines for more information: http://journals.plos.org/plosone/s/supporting-information.

Additional Editor Comments:

Dear Mr Callahan - Please make sure that reviewer 2's insightful comments are all adequately addressed given the importance of the topic and that of the analyses in this case.

[Note: HTML markup is below. Please do not edit.]

Reviewers' comments:

Reviewer's Responses to Questions

**Comments to the Author**

1. Is the manuscript technically sound, and do the data support the conclusions?

Reviewer #1: Yes

Reviewer #2: Partly

**********

2. Has the statistical analysis been performed appropriately and rigorously?

Reviewer #1: Yes

Reviewer #2: Yes

**********

3. Have the authors made all data underlying the findings in their manuscript fully available?

The PLOS Data policy

Reviewer #1: Yes

Reviewer #2: No

**********

4. Is the manuscript presented in an intelligible fashion and written in standard English?

Reviewer #1: Yes

Reviewer #2: Yes

**********

Reviewer #1: This article is extremely informative and innovative in its use of modeling formulas, particularly highlighting some limitations in prior work focusing on FGC. It is well written and referenced, and still an important topic world-wide.

One question did occur to me in terms of migration timing and the impact of selective migration. Looking at involuntary mobility, might the formula need adjusting when assessing women and girls held in refugee camps for many years? There are refugee camps where migrant groups may be very heterogeneous or may be influenced by outside factors over time. Another question is whether women and girls seeking asylum fit into the refugee involuntary migrant group.

The article held my interest laid a further foundation for work in this field.

Reviewer #2: Overall assessment

The manuscript addresses an important and policy-relevant methodological problem: how to estimate FGM/C prevalence and risk among immigrant and diaspora populations while accounting for migration timing, selective migration, and post-migration changes in exposure. The paper is ambitious and potentially valuable, especially in its attempt to distinguish between women and girls already living with FGM/C and girls who remain at future risk.

However, the manuscript requires substantial revision before it can be properly evaluated for publication. The literature review and conceptual framing contain several substantive inaccuracies, including mischaracterizations of the methods used in key previous studies. There is also repeated conceptual and methodological confusion between methods used for prevalence estimation and for risk estimation. These are related and have many aspects in common but are distinct, and methods have been developed separately: the manuscript does not always distinguish them clearly.

In addition, several of the proposed methodological innovations are introduced at a conceptual level but are not estimated, not operationalized, and not accompanied by a clear explanation of how they could be implemented in practice. This weakens the practical applicability of the model and makes parts of the paper appear overly abstract.

The paper would be stronger if it were more focused. I would encourage the author to drop from the paper all that is not feasible, and retain and develop only those components that are actually implemented in the empirical calculation, removing or clearly relegating to a short future research section those elements that are not currently applicable.

Major comments

(1) The literature review requires clarification, as it currently tends to conflate studies with different objects of estimation and different methodological approaches.

The manuscript states that the “Extrapolation-of-FGM/C-Countries’ Prevalence-Data method” was developed by Yoder et al. and Van Baelen et al. and later codified by EIGE. This formulation is misleading. Yoder et al. and Van Baelen et al. primarily estimate FGM/C prevalence among women and girls, whereas EIGE’s step-by-step guide focuses specifically on estimating girls at risk of FGM/C.

Relatedly, the manuscript attributes to Yoder et al. and Van Baelen et al. a modeling structure that includes a migration and acculturation parameter, m. This does not appear to be accurate. In particular, Van Baelen et al. do not use such a coefficient. Van Baelen said that “the estimations provided for this study follow the standard for baseline estimation proposed by EIGE” (that does not include m) and never mention acculturation, neither does Yoder.

This is not a minor terminological issue: it affects the way the manuscript reconstructs the existing literature and the extent to which the proposed model can be presented as an extension of previous work.

The author should revise the literature review to distinguish more carefully between:

prevalence estimation among women and girls;

estimation of girls currently or prospectively at risk;

At present, the review risks misrepresenting the methodological lineage of the field.

The use of m and the median age for cutting is typical of the EIGE approach that explicitly refers to risk.

(2) The use of a generalized gamma distribution to model the timing of FGM/C is one of the most interesting methodological innovations in the manuscript. It is preferable to fixed age-threshold approaches, because age at FGM/C varies substantially across countries and communities. However, the fitting procedure is not described in sufficient detail for replication. The manuscript states that the parameters were estimated through a three-stage process involving analytical root-finding, dense numerical search, and bounded nonlinear optimization. This is not enough for readers to evaluate the robustness of the estimates.

The author should provide:

the exact objective function used;

parameter constraints;

starting values or search ranges;

software or packages used;

goodness-of-fit criteria;

treatment of countries with quartiles equal or close to zero;

code or pseudocode in the supplementary material.

Without these details, the generalized gamma estimates remain difficult to assess.

The gamma-based approach assumes a unimodal distribution of age at FGM/C. The author acknowledges that some countries may have wider or potentially multimodal distributions, including Liberia. However, this issue deserves more attention because the paper’s main empirical conclusion depends heavily on the shape of the age-at-cutting distribution.

The author should conduct sensitivity analyses and provide then in the appendix.

(3) The expanded model includes several factors, including the Migration Selection Factor, Ethno-religious Loading Factor, Prevention Impact Factor, Circular Migration Risk Factor, and Acculturation Impact Factor. Conceptually, these dimensions may be relevant. Empirically, however, several are not implemented.

For example, the Ethno-religious Loading Factor is set to 1, although the manuscript argues that ethno-religious selection could be very important. The Circular Migration Risk Factor is conceptualized but not quantified. The Acculturation Impact Factor is set to zero because the paper does not offer a method to estimate it.

As several proposed innovations are not estimated, and in some cases it is not even clear what data would be required or how the parameters could be operationalized this raises a fundamental question: if the paper does not offer a method for estimating a parameter, what is the analytical value of including it in the model? The sentence “Since this paper does not offer a method by which to estimate the acculturation effect, the AIF was set accordingly (AIF = 0)” is particularly problematic. If the author cannot explain how the acculturation effect should be estimated, why should it be part of the formal model?

At present, the model risks appearing more empirically complete than it actually is.

If the author wishes to retain these additional parameters, the manuscript should clearly distinguish between:

components actually estimated in the present analysis;

components included only as theoretical extensions;

components explored through sensitivity analysis.

And provide a clear assessment of:

what data would be required;

whether such data currently exist;

how the parameters could be estimated;

why they are not implemented in the present study;

how sensitive the results would be to plausible values;

worked examples, even using hypothetical or simulated data.

Otherwise, these components should be removed from the main model and discussed only briefly as possible directions for future research. In their current form, they risk remaining pure abstraction without a clear horizon of empirical feasibility.

(4) The Prevention Impact Factor is linked to criminological concepts such as labelling, deterrence, desistance, and community abandonment. This is an interesting theoretical framing. However, the numerical values assigned to these scenarios—0, 0.25, 0.50, and 0.75—appear arbitrary.

The author should present these values explicitly as hypothetical scenarios rather than empirically grounded estimates. The sensitivity analysis is useful, but the interpretation should be cautious, especially because the PIF substantially affects the estimated number of girls still at risk.

(5) The manuscript should revise its terminology around selective migration. Ortensi et al. do not refer to a “Migration Selection Factor” in the way the manuscript suggests. Rather, they discuss a correction according to the selection hypothesis. The manuscript should either use Ortensi et al.’s terminology or explicitly state that “Migration Selection Factor” is the author’s own label for this correction.

(6) The manuscript argues that previous estimates overstate the number of girls at risk and understate the number of women and girls already living with FGM/C. This is a potentially important finding. However, because this is driven mainly by assumptions and scenarios that cannot be verified the policy conclusion should be presented with greater attention to uncertainty.

Other minor comments

The distinction between demographic and ethnic selection should also be reconsidered. The statement that “selective migration is not only a factor of demographics, but it also shapes the ethnic composition of the immigrant population” is not entirely convincing, since ethnic composition, when mediated by migration patterns, is itself a demographic characteristic of the migrant population.

the Nigerian example: The manuscript interprets higher FGM/C prevalence among Nigerian migrants mainly as evidence of ethnic selective migration. However, at least in the Italian case, this pattern is also strongly related to geographical selection, as many Nigerian migrants come from Edo State. The manuscript should therefore avoid framing the Nigerian example only in ethnic terms and should acknowledge that regional/geographical selection may be equally or more relevant in some destination countries.

The manuscript should clarify why the generalized gamma distribution was preferred over simpler alternatives, such as empirical cumulative distributions based on DHS/MICS age categories.

The terminology “age-desegregated” appears to be used where “age-disaggregated” may be intended.

The author should review the use of terms such as “prevalence,” “risk,” “impacted,” “living with,” and “at risk” to ensure that they are used consistently throughout the paper.

**********

what does this mean?). If published, this will include your full peer review and any attached files.

If you choose “no”, your identity will remain anonymous but your review may still be made public.

**Do you want your identity to be public for this peer review?**  For information about this choice, including consent withdrawal, please see our Privacy Policy

Reviewer #1: No

Reviewer #2: No

**********

[NOTE: If reviewer comments were submitted as an attachment file, they will be attached to this email and accessible via the submission site. Please log into your account, locate the manuscript record, and check for the action link "View Attachments". If this link does not appear, there are no attachment files.]

To ensure your figures meet our technical requirements, please review our figure guidelines: https://journals.plos.org/plosone/s/figures

You may also use PLOS’s free figure tool, NAAS, to help you prepare publication quality figures: https://journals.plos.org/plosone/s/figures#loc-tools-for-figure-preparation.

NAAS will assess whether your figures meet our technical requirements by comparing each figure against our figure specifications.

# Author comment: Author response to Decision Letter 1

**DOI:** 10.1371/journal.pone.0360038.r002

25 Jun 2026

I have uploaded a comprehensive response to reviewer document

Attachment Submitted filename: Response.docx

# Peer review: Decision Letter 1

**DOI:** 10.1371/journal.pone.0360038.r003

**Authors:** Surajit Sen Surajit Sen

29 Jul 2026

Dear Dr. Callaghan,

Thank you for submitting your manuscript to PLOS ONE. After careful consideration, we feel that it has merit but does not fully meet PLOS ONE’s publication criteria as it currently stands. Therefore, we invite you to submit a revised version of the manuscript that addresses the points raised during the review process.

1) The introduction of the Ethno-religious Loading Factor remains insufficiently justified. The revised manuscript applies an ELF of 2 to the Nigerian population and 0.5 to Egyptian girls growing up in the diaspora. However, these values appear to be selected on an ad hoc basis rather than derived from a transparent and reproducible estimation procedure.

I therefore recommend one of these solutions

- either removing ELF from the main estimates and presenting it only as part of a clearly labelled sensitivity analysis.

The manuscript should also state more explicitly that the current ELF values are illustrative assumptions rather than empirically validated parameters.

2) The manuscript still does not distinguish consistently between women and girls living with FGM/C, girls at risk of FGM/C, and the broader population described as “impacted.” This is particularly evident in Table 1, where the sum of those estimated to be living with FGM/C and those estimated to be at risk is reported as “estimated FGM/C prevalence.”

This terminology is not conceptually correct. Girls who are currently at risk have not necessarily undergone FGM/C and may never do so. Therefore, the combined category of “living with FGM/C” plus “at risk” cannot be interpreted as prevalence.

It would be more appropriate to report separately:

-estimated prevalence of FGM/C;

-estimated number or proportion currently at risk;

-total population potentially impacted, if the authors wish to retain this broader category

3) Relatedly, expressions such as “FGM/C that occurred while US-based” are too definitive, because the model does not observe whether FGM/C actually occurred after migration.

It probabilistically allocates expected cases across pre-migration and post-migration periods. The results should therefore be described consistently as model-based estimates or expected numbers rather than observed events.

Academic Editor Comments -

Please submit your revised manuscript by Sep 12 2026 11:59PM. If you will need more time than this to complete your revisions, please reply to this message or contact the journal office at plosone@plos.org. When you're ready to submit your revision, log on to https://www.editorialmanager.com/pone/ and select the 'Submissions Needing Revision' folder to locate your manuscript file.

- A letter that responds to each point raised by the academic editor and reviewer(s). You should upload this letter as a separate file labeled 'Response to Reviewers'.
- A marked-up copy of your manuscript that highlights changes made to the original version. You should upload this as a separate file labeled 'Revised Manuscript with Track Changes'.
- An unmarked version of your revised paper without tracked changes. You should upload this as a separate file labeled 'Manuscript'.

If you would like to make changes to your financial disclosure, please include your updated statement in your cover letter. Guidelines for resubmitting your figure files are available below the reviewer comments at the end of this letter.

If applicable, we recommend that you deposit your laboratory protocols in protocols.io to enhance the reproducibility of your results. Protocols.io assigns your protocol its own identifier (DOI) so that it can be cited independently in the future. For instructions see: https://journals.plos.org/plosone/s/submission-guidelines#loc-laboratory-protocols. Additionally, PLOS ONE offers an option for publishing peer-reviewed Lab Protocol articles, which describe protocols hosted on protocols.io. Read more information on sharing protocols at https://plos.org/protocols?utm_medium=editorial-email&utm_source=authorletters&utm_campaign=protocols.

As the corresponding author, your ORCID iD is verified in the submission system and will appear in the published article. PLOS supports the use of ORCID, and we encourage all coauthors to register for an ORCID iD and use it as well. Please encourage your coauthors to verify their ORCID iD within the submission system before final acceptance, as unverified ORCID iDs will not appear in the published article. *Only*  the individual author can complete the verification step; PLOS staff *cannot*  verify ORCID iDs on behalf of authors.

We look forward to receiving your revised manuscript.

Kind regards,

Surajit Sen, PhD

Academic Editor

PLOS One

Journal Requirements:

If the reviewer comments include a recommendation to cite specific previously published works, please review and evaluate these publications to determine whether they are relevant and should be cited. There is no requirement to cite these works unless the editor has indicated otherwise.

Please review your reference list to ensure that it is complete and correct. If you have cited papers that have been retracted, please include the rationale for doing so in the manuscript text, or remove these references and replace them with relevant current references. Any changes to the reference list should be mentioned in the rebuttal letter that accompanies your revised manuscript. If you need to cite a retracted article, indicate the article’s retracted status in the References list and also include a citation and full reference for the retraction notice.

Additional Editor Comments :

Dear Mr Callahan -

Thank you for the significant revision that you have incorporated in this revised version. I would request you to study in detail the few concerns of and detailed suggestions made by Reviewer 2. Please address these suggestions in a second revision.

Thank you again for your careful attention to the suggested changes in the previous version.

Best wishes,

Surajit Sen, Professor of Physics, SUNY Buffalo

Academic Editor

[Note: HTML markup is below. Please do not edit.]

Reviewers' comments:

Reviewer's Responses to Questions

**Comments to the Author**

Reviewer #2: (No Response)

**********

2. Is the manuscript technically sound, and do the data support the conclusions?

Reviewer #2: Yes

**********

3. Has the statistical analysis been performed appropriately and rigorously?

Reviewer #2: Yes

**********

4. Have the authors made all data underlying the findings in their manuscript fully available?

The PLOS Data policy

Reviewer #2: Yes

**********

5. Is the manuscript presented in an intelligible fashion and written in standard English?

Reviewer #2: Yes

**********

Reviewer #2: The revised manuscript has improved substantially, and most of the concerns raised in the previous review have been addressed. However, two important issues remain unresolved and should be clarified before the manuscript can be published

1) The introduction of the Ethno-religious Loading Factor remains insufficiently justified. The revised manuscript applies an ELF of 2 to the Nigerian population and 0.5 to Egyptian girls growing up in the diaspora. However, these values appear to be selected on an ad hoc basis rather than derived from a transparent and reproducible estimation procedure.

For Nigeria, the doubling factor is inferred from a small number of local studies conducted in different destination countries and among populations that may not be representative of Nigerian migrants in the United States. For Egypt, differences in attitudes towards continuation of FGM/C are translated directly into a 50% reduction in risk, although attitudinal differences cannot automatically be interpreted as equivalent differences in behavioural prevalence or incidence.

More importantly, no general procedure is provided for estimating ELF across origin groups, and no sensitivity analysis is presented to show how the results change under alternative plausible values.

I therefore recommend one of these solutions

- Keeping ELF and 1) acknowledging the limitations 2) presenting estimates with and without ELF and assessing clearly the impact of inserting such a speculative coefficient

- either removing ELF from the main estimates and presenting it only as part of a clearly labelled sensitivity analysis.

The manuscript should also state more explicitly that the current ELF values are illustrative assumptions rather than empirically validated parameters.

2) The manuscript still does not distinguish consistently between women and girls living with FGM/C, girls at risk of FGM/C, and the broader population described as “impacted.” This is particularly evident in Table 1, where the sum of those estimated to be living with FGM/C and those estimated to be at risk is reported as “estimated FGM/C prevalence.”

This terminology is not conceptually correct. Girls who are currently at risk have not necessarily undergone FGM/C and may never do so. Therefore, the combined category of “living with FGM/C” plus “at risk” cannot be interpreted as prevalence.

It would be more appropriate to report separately:

-estimated prevalence of FGM/C;

-estimated number or proportion currently at risk;

-total population potentially impacted, if the authors wish to retain this broader category

3) Relatedly, expressions such as “FGM/C that occurred while US-based” are too definitive, because the model does not observe whether FGM/C actually occurred after migration.

It probabilistically allocates expected cases across pre-migration and post-migration periods. The results should therefore be described consistently as model-based estimates or expected numbers rather than observed events.

These distinctions are essential because the current terminology risks overstating the precision and empirical status of the findings.

**********

what does this mean?). If published, this will include your full peer review and any attached files.

If you choose “no”, your identity will remain anonymous but your review may still be made public.

**Do you want your identity to be public for this peer review?**  For information about this choice, including consent withdrawal, please see our Privacy Policy

Reviewer #2: No

**********

[NOTE: If reviewer comments were submitted as an attachment file, they will be attached to this email and accessible via the submission site. Please log into your account, locate the manuscript record, and check for the action link "View Attachments". If this link does not appear, there are no attachment files.]

To ensure your figures meet our technical requirements, please review our figure guidelines: https://journals.plos.org/plosone/s/figures

You may also use PLOS’s free figure tool, NAAS, to help you prepare publication quality figures: https://journals.plos.org/plosone/s/figures#loc-tools-for-figure-preparation.

NAAS will assess whether your figures meet our technical requirements by comparing each figure against our figure specifications.

# Author comment: Author response to Decision Letter 2

**DOI:** 10.1371/journal.pone.0360038.r004

31 Jul 2026

I have included a Response to Reviewers note which details all of the responses

Attachment Submitted filename: Response (v2).docx

# Peer review: Decision Letter 2

**DOI:** 10.1371/journal.pone.0360038.r005

**Authors:** Surajit Sen Surajit Sen

22 Sep 2026

Modelling the impact of migration on FGM/C prevalence and risk in immigrant and diaspora populations

PONE-D-26-15067R2

Dear Dr. Callaghan,

We’re pleased to inform you that your manuscript has been judged scientifically suitable for publication and will be formally accepted for publication once it meets all outstanding technical requirements.

Within one week, you’ll receive an e-mail detailing the required amendments. When these have been addressed, you’ll receive a formal acceptance letter and your manuscript will be scheduled for publication.

An invoice will be generated when your article is formally accepted. Please note, if your institution has a publishing partnership with PLOS and your article meets the relevant criteria, all or part of your publication costs will be covered. Please make sure your user information is up-to-date by logging into Editorial Manager at Editorial Manager® and clicking the ‘Update My Information' link at the top of the page. For questions related to billing, please contact billing support.

If your institution or institutions have a press office, please notify them about your upcoming paper to help maximize its impact. If they’ll be preparing press materials, please inform our press team as soon as possible -- no later than 48 hours after receiving the formal acceptance. Your manuscript will remain under strict press embargo until 2 pm Eastern Time on the date of publication. For more information, please contact onepress@plos.org.

Kind regards,

Surajit Sen, PhD

Academic Editor

PLOS One

Additional Editor Comments (optional):

Reviewers' comments:

Reviewer's Responses to Questions

**Comments to the Author**

Reviewer #2: All comments have been addressed

**********

2. Is the manuscript technically sound, and do the data support the conclusions?

Reviewer #2: Yes

**********

3. Has the statistical analysis been performed appropriately and rigorously?

Reviewer #2: No

**********

4. Have the authors made all data underlying the findings in their manuscript fully available?

The PLOS Data policy

Reviewer #2: Yes

**********

5. Is the manuscript presented in an intelligible fashion and written in standard English?

Reviewer #2: Yes

**********

Reviewer #2: All revision have been addressed, the manuscript can be now published in its current form

**********

what does this mean?). If published, this will include your full peer review and any attached files.

If you choose “no”, your identity will remain anonymous but your review may still be made public.

**Do you want your identity to be public for this peer review?**  For information about this choice, including consent withdrawal, please see our Privacy Policy

Reviewer #2: No

**********

# Editor report: Acceptance letter

**DOI:** 10.1371/journal.pone.0360038.r006

**Authors:** Surajit Sen Surajit Sen

PONE-D-26-15067R2

PLOS One

Dear Dr. Callaghan,

I'm pleased to inform you that your manuscript has been deemed suitable for publication in PLOS One. Congratulations! Your manuscript is now being handed over to our production team.

At this stage, our production department will prepare your paper for publication. This includes ensuring the following:

* All references, tables, and figures are properly cited

* All relevant supporting information is included in the manuscript submission,

* There are no issues that prevent the paper from being properly typeset

You will receive further instructions from the production team, including instructions on how to review your proof when it is ready. Please keep in mind that we are working through a large volume of accepted articles, so please give us a few days to review your paper and let you know the next and final steps.

Lastly, if your institution or institutions have a press office, please let them know about your upcoming paper now to help maximize its impact. If they'll be preparing press materials, please inform our press team within the next 48 hours. Your manuscript will remain under strict press embargo until 2 pm Eastern Time on the date of publication. For more information, please contact onepress@plos.org.

You will receive an invoice from PLOS for your publication fee after your manuscript has reached the completed accept phase. If you receive an email requesting payment before acceptance or for any other service, this may be a phishing scheme. Learn how to identify phishing emails and protect your accounts at https://explore.plos.org/phishing.

If we can help with anything else, please email us at customercare@plos.org.

Thank you for submitting your work to PLOS One and supporting open access.

Kind regards,

PLOS One Editorial Office Staff

on behalf of

Dr. Surajit Sen Surajit Sen

Academic Editor

PLOS One
